# Supplementary material for: Cassava brown streak virus has a rapidly evolving genome: implications for virus speciation, variability, diagnosis and host resistance
Source: Sci Rep. 2016 Nov 3;6:36164. doi: 10.1038/srep36164 (PMC5093738; doi:10.1038/srep36164)
Supplement: Supplementary Information [file srep36164-s1.pdf]

Supplemental Figures 1 and 2

Cassava brown streak virus has a rapidly evolving genome: implications for virus speciation, variability, diagnosis and host resistance

Titus Alicai, Joseph Ndunguru, Peter Sseruwagi, Fred Tairo, Geoffrey Okao-Okuja, Resty Nanvubya, Lilliane Kiiza, Laura Kubatko, Monica A. Kehoe and Laura M. Boykin

Supplemental Figure 1. CBSV Amino Acid variability obtained using datamonkey.org. Once files are uploaded to the site, the images below are obtained from the “Information from upload” tab and the pdf is downloaded.

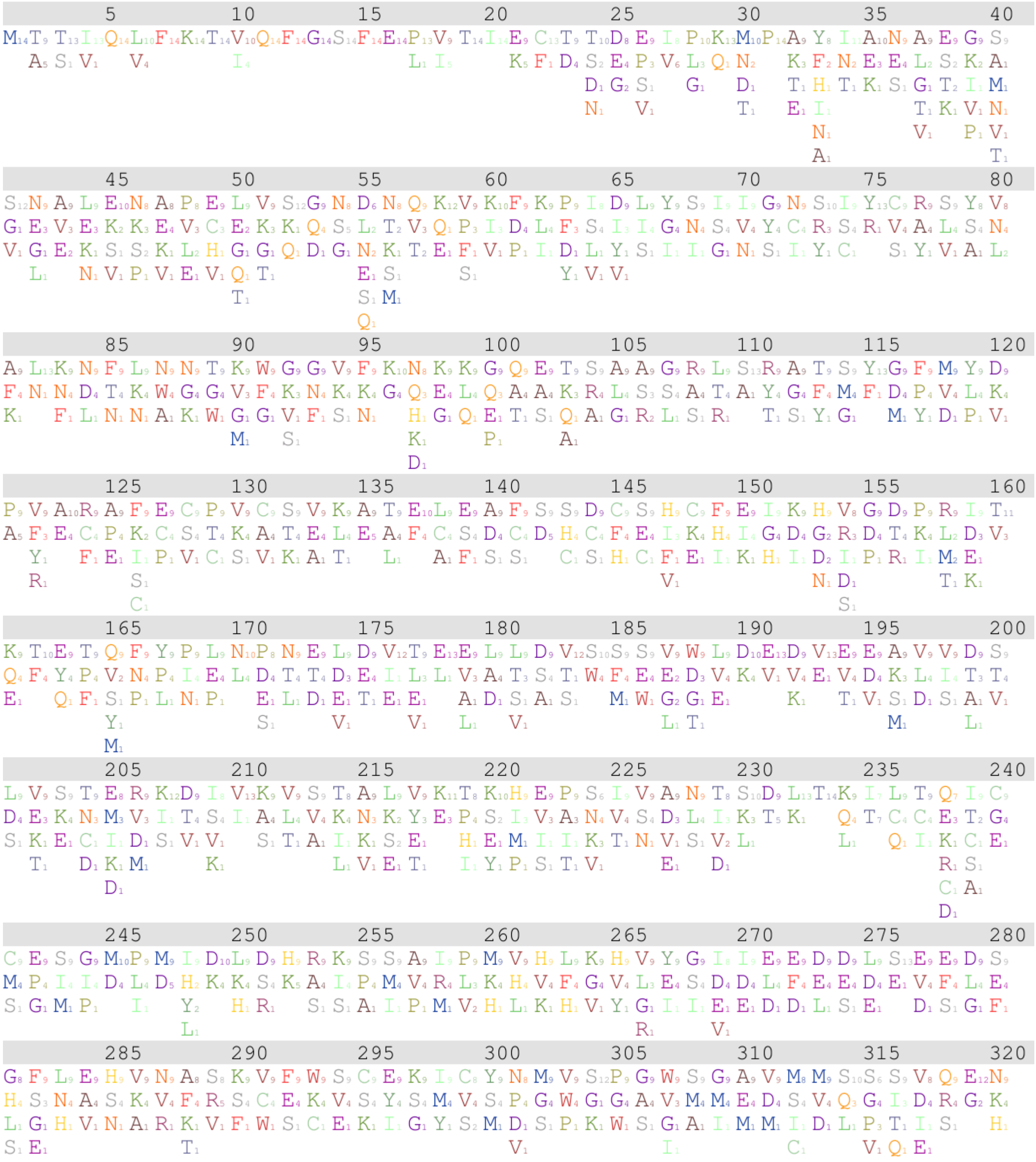

				325					330					335					340					345					350					355					360
E_{9}	H_{13}	D_{9}	M_{9}	F_{10}	D_{9}	F_{9}	V_{9}	D_{9}	D_{9}	M_{9}	C_{9}	V_{9}	V_{9}	Q_{9}	G_{9}	K_{10}	N_{9}	K_{9}	I_{12}	S_{9}	G_{9}	K_{9}	I_{9}	E_{9}	N_{9}	A_{9}	L_{9}	V_{9}	S_{10}	K_{9}	S_{9}	W_{9}	D_{9}	E_{9}	L_{9}	Q_{9}	Q_{6}	I_{9}	E_{9}
F_{4}	M_{1}	F_{5}	I_{2}	D_{4}	G_{4}	M_{4}	C_{4}	V_{4}	V_{4}	Q_{4}	G_{4}	K_{4}	N_{4}	K_{4}	S_{3}	S_{3}	G_{4}	R_{4}	G_{1}	E_{4}	N_{4}	A_{4}	L_{4}	V_{4}	L_{3}	K_{4}	S_{5}	S_{2}	E_{3}	D_{3}	L_{4}	S_{3}	E_{4}	I_{4}	E_{4}	L_{4}	Y_{4}	S_{4}	F_{4}
D_{1}			V_{2}		V_{1}	N_{1}	D_{1}	M_{1}	C_{1}	V_{1}	V_{1}	Q_{1}	G_{1}	E_{1}	E_{1}	N_{1}	I_{1}	S_{1}	V_{1}	K_{1}	I_{1}	E_{1}	N_{1}	A_{1}	P_{2}	V_{1}		K_{1}	D_{1}	W_{1}	D_{1}	G_{1}	L_{1}	Q_{1}	Q_{1}	I_{1}	H_{3}	M_{1}	Y_{1}
			N_{1}												N_{1}													N_{1}		E_{1}		E_{1}					E_{1}		
																												D_{1}											

				365					370					375					380					385					390					395					400
M_{9}	Y_{9}	S_{13}	F_{9}	D_{9}	L_{9}	S_{9}	W_{9}	A_{9}	R_{10}	S_{9}	R_{9}	D_{8}	D_{9}	F_{13}	F_{9}	K_{8}	Y_{9}	F_{9}	D_{9}	E_{9}	D_{9}	V_{9}	G_{9}	S_{9}	L_{9}	I_{9}	S_{9}	T_{9}	C_{9}	C_{9}	T_{9}	P_{9}	S_{8}	T_{9}	L_{10}	W_{9}	L_{9}	Y_{9}	A_{9}
D_{4}	L_{4}	D_{1}	W_{4}	A_{4}	R_{4}	T_{4}	R_{5}	D_{4}	N_{4}	F_{4}	F_{3}	R_{4}	H_{4}	K_{1}	D_{4}	D_{4}	D_{5}	V_{4}	G_{4}	K_{4}	L_{4}	I_{4}	S_{4}	T_{4}	C_{4}	C_{4}	T_{4}	P_{4}	S_{4}	T_{4}	L_{4}	W_{4}	L_{5}	Y_{4}	A_{4}	K_{4}	K_{4}	A_{4}	S_{4}
S_{1}	F_{1}		L_{1}	S_{1}	W_{1}	A_{1}		S_{1}		D_{1}	D_{1}	F_{1}	F_{1}		Y_{1}	F_{1}		E_{1}	N_{1}	V_{1}	S_{1}	S_{1}	L_{1}	I_{1}	S_{1}	T_{1}	C_{1}	C_{1}	T_{1}	P_{1}	S_{1}	T_{1}	T_{1}	W_{1}		Y_{1}	A_{1}	K_{1}	K_{1}
											Y_{1}	N_{1}																											

				405					410					415					420					425					430					435					440
K_{9}	K_{9}	A_{9}	S_{9}	F_{9}	Y_{10}	K_{9}	Y_{9}	V_{9}	D_{9}	H_{8}	M_{9}	I_{9}	L_{9}	K_{9}	G_{9}	S_{9}	P_{9}	L_{13}	V_{13}	D_{9}	I_{9}	L_{9}	V_{9}	K_{9}	M_{9}	E_{9}	Y_{9}	V_{9}	G_{9}	K_{8}	H_{9}	L_{9}	E_{9}	M_{9}	F_{9}	N_{9}	S_{9}	V_{9}	E_{9}
F_{4}	Y_{4}	K_{4}	Y_{5}	V_{4}	D_{4}	H_{4}	M_{4}	M_{4}	L_{4}	K_{4}	G_{4}	S_{4}	P_{4}	L_{4}	V_{4}	D_{4}	M_{4}	D_{1}	I_{1}	K_{4}	M_{4}	E_{4}	Y_{4}	V_{4}	G_{4}	K_{4}	H_{4}	L_{4}	E_{4}	M_{4}	F_{4}	N_{4}	S_{4}	V_{4}	E_{4}	D_{4}	V_{4}	C_{4}	L_{4}
A_{1}	S_{1}	F_{1}		K_{1}		V_{1}	D_{1}	H_{1}	M_{1}	I_{1}	L_{1}	K_{1}	G_{1}	S_{1}	P_{1}	L_{1}	V_{1}			L_{1}	V_{1}	K_{1}	M_{1}	E_{1}	Y_{1}	V_{1}	G_{1}	K_{1}	H_{1}	L_{2}	E_{1}	M_{1}	F_{1}	N_{1}	S_{1}	V_{1}	E_{1}	D_{1}	V_{1}

				445					450					455					460					465					470					475					480
D_{9}	V_{9}	C_{9}	L_{9}	E_{9}	Y_{9}	A_{9}	H_{9}	F_{9}	M_{9}	Q_{13}	D_{9}	L_{9}	M_{9}	Q_{9}	D_{13}	H_{8}	V_{9}	T_{8}	D_{9}	Q_{8}	S_{9}	N_{8}	E_{9}	D_{8}	I_{9}	L_{9}	R_{9}	V_{9}	K_{9}	N_{9}	L_{9}	I_{9}	R_{9}	S_{13}	Y_{9}	F_{9}	D_{9}	S_{13}	V_{9}
E_{4}	Y_{4}	A_{4}	H_{4}	F_{4}	M_{4}	Q_{4}	D_{4}	M_{2}	I_{4}	L_{1}	E_{4}	Q_{5}	V_{3}	N_{4}	V_{1}	Q_{5}	S_{4}	D_{4}	E_{4}	N_{4}	M_{4}	M_{4}	R_{4}	V_{4}	K_{4}	N_{4}	L_{4}	I_{4}	R_{4}	S_{4}	Y_{4}	F_{4}	D_{4}	F_{1}	V_{4}	I_{3}	E_{4}	I_{1}	N_{4}
C_{1}	L_{1}	E_{1}	Y_{1}	A_{1}	H_{1}	F_{1}	M_{1}	V_{2}	D_{1}		M_{1}		I_{1}	C_{1}		T_{1}	D_{1}	Q_{1}	S_{1}	H_{1}	E_{1}	D_{1}	I_{1}	L_{1}	R_{1}	V_{1}	K_{1}	N_{1}	L_{1}	I_{1}	R_{1}	S_{1}	Y_{1}		D_{1}	S_{1}	V_{1}		E_{1}
								Q_{1}					D_{1}					N_{1}		S_{1}		T_{1}		N_{1}												M_{1}			

				485					490					495					500					505					510					515					520
I_{9}	E_{9}	A_{9}	N_{8}	K_{9}	Y_{9}	E_{9}	L_{9}	I_{13}	D_{12}	R_{9}	I_{9}	I_{9}	D_{8}	K_{9}	K_{9}	T_{8}	Q_{13}	L_{9}	E_{9}	A_{9}	Q_{9}	E_{9}	I_{9}	I_{9}	S_{9}	R_{9}	E_{9}	L_{9}	I_{9}	K_{9}	H_{9}	Q_{9}	Y_{9}	G_{9}	A_{9}	L_{9}	F_{9}	S_{9}	L_{13}
K_{4}	Y_{4}	E_{4}	L_{4}	I_{4}	D_{4}	S_{4}	I_{4}	R_{1}	I_{1}	K_{4}	K_{4}	S_{4}	E_{5}	L_{4}	E_{4}	A_{4}	E_{1}	E_{4}	V_{4}	I_{4}	S_{4}	R_{4}	E_{4}	L_{4}	I_{4}	K_{3}	H_{4}	Q_{4}	Y_{4}	G_{4}	D_{4}	L_{4}	F_{4}	S_{4}	L_{4}	R_{4}	E_{4}	R_{5}	E_{1}
A_{1}	N_{1}	K_{1}	S_{1}	E_{1}	L_{1}	I_{1}	D_{1}		E_{1}	I_{1}	E_{1}	K_{1}	K_{1}	T_{1}	Q_{1}	L_{1}		A_{1}	Q_{1}	E_{1}	I_{1}	I_{1}	S_{1}	R_{1}	E_{1}	L_{1}	I_{1}	K_{1}	H_{1}	Q_{1}	Y_{1}	G_{1}	A_{1}	L_{1}	F_{1}	S_{1}	L_{1}		
			Y_{1}													S_{1}										S_{1}													

				525					530					535					540					545					550					555					560
R_{10}	E_{9}	R_{9}	I_{14}	S_{9}	I_{9}	N_{9}	I_{9}	G_{9}	F_{9}	D_{9}	S_{14}	A_{9}	S_{10}	L_{9}	S_{11}	S_{9}	L_{9}	W_{9}	K_{9}	E_{10}	S_{9}	E_{9}	S_{8}	L_{9}	S_{13}	R_{9}	S_{10}	D_{9}	S_{13}	S_{9}	F_{9}	F_{9}	S_{13}	C_{9}	L_{9}	M_{9}	S_{9}	S_{9}	P_{9}
L_{4}	I_{4}	N_{4}		G_{4}	F_{4}	D_{4}	S_{4}	A_{4}	K_{4}	L_{4}		S_{4}	L_{4}	W_{4}	E_{1}	E_{4}	S_{4}	E_{5}	S_{5}	V_{4}	K_{4}	Q_{4}	K_{6}	S_{3}	K_{1}	S_{4}	I_{2}	I_{4}	I_{1}	C_{4}	L_{4}	I_{4}	L_{1}	K_{4}	P_{4}	G_{4}	M_{4}	E_{4}	M_{5}
	M_{1}	S_{1}		N_{1}	V_{1}	G_{1}	F_{1}	D_{1}	S_{1}	A_{1}		L_{1}		S_{1}	L_{1}	W_{1}	K_{1}				M_{1}	L_{1}		G_{1}		D_{1}	V_{2}	S_{1}		F_{1}	S_{1}	C_{1}		M_{1}	S_{1}	S_{1}	P_{1}	G_{1}	
															Q_{1}									R_{1}															

				565					570					575					580					585					590					595					600
G_{9}	M_{10}	E_{9}	M_{9}	C_{9}	A_{9}	N_{8}	W_{9}	I_{8}	L_{8}	S_{8}	V_{9}	C_{9}	S_{14}	S_{9}	S_{10}	Y_{9}	S_{9}	N_{9}	T_{9}	L_{9}	Y_{9}	L_{9}	V_{9}	D_{9}	K_{8}	G_{9}	V_{9}	V_{8}	F_{9}	F_{9}	W_{9}	S_{13}	S_{9}	M_{5}	V_{9}	H_{9}	I_{9}	F_{9}	Q_{9}
L_{3}	A_{4}	N_{4}	W_{4}	I_{4}	S_{4}	E_{4}	V_{3}	F_{4}	K_{4}	N_{4}	C_{4}	Y_{4}		N_{4}	T_{4}	M_{4}	Y_{4}	L_{5}	V_{4}	D_{4}	R_{4}	S_{4}	M_{4}	M_{4}	F_{4}	I_{4}	W_{4}	N_{4}	R_{4}	M_{4}	V_{4}	V_{1}	I_{4}	F_{4}	N_{4}	S_{4}	C_{4}	V_{4}	Y_{4}
F_{1}		C_{1}	A_{1}	N_{1}	W_{1}	T_{1}	A_{1}	T_{1}	S_{1}	K_{1}	S_{1}	S_{1}		Y_{1}		N_{1}	I_{1}		Y_{1}	M_{1}	V_{1}	D_{1}	K_{1}	G_{1}	V_{1}	V_{1}	F_{1}	F_{1}	W_{1}	S_{1}	S_{1}		V_{1}	V_{4}	I_{1}	F_{1}	Q_{1}	T_{1}	C_{1}
E_{1}						S_{1}	L_{1}	S_{1}	V_{1}	C_{1}															S_{1}			I_{1}						H_{1}					

				605					610					615					620					625					630					635					640
T_{9}	C_{9}	V_{9}	Y_{10}	G_{9}	Y_{9}	W_{9}	N_{9}	L_{9}	W_{9}	F_{9}	S_{9}	K_{9}	A_{9}	V_{10}	C_{9}	V_{9}	L_{9}	M_{9}	A_{9}	F_{9}	V_{9}	I_{9}	M_{9}	G_{9}	F_{9}	S_{9}	G_{9}	K_{9}	A_{5}	M_{9}	D_{9}	Y_{9}	L_{9}	K_{13}	K_{9}	L_{9}	I_{8}	K_{13}	N_{9}
G_{4}	Y_{5}	W_{4}	N_{4}	L_{4}	W_{4}	F_{4}	R_{4}	K_{4}	A_{4}	V_{3}	C_{4}	V_{5}	L_{4}	M_{4}	V_{4}	F_{4}	V_{3}	V_{4}	T_{4}	G_{4}	F_{4}	S_{4}	S_{4}	K_{3}	I_{4}	I_{4}	E_{3}	F_{4}	L_{4}	K_{3}	K_{4}	L_{4}	I_{3}	L_{1}	N_{4}	E_{4}	S_{4}	E_{1}	Q_{4}
V_{1}		G_{1}		W_{1}	N_{1}	L_{1}	W_{1}	F_{1}	S_{1}	M_{1}	A_{1}		C_{1}		L_{1}	M_{1}	A_{1}	F_{1}	V_{1}	I_{1}	M_{1}	G_{1}	F_{1}	S_{2}	G_{1}	K_{1}	V_{1}	M_{1}	V_{4}	Y_{1}	L_{1}	K_{1}	M_{1}		I_{1}	K_{1}	N_{1}		S_{1}
										K_{1}							M_{1}										D_{1}		D_{1}	S_{1}			K_{1}				M_{1}		

				645					650					655					660					665					670					675					680
E_{9}	S_{9}	K_{10}	Q_{9}	A_{9}	I_{9}	Q_{9}	Y_{9}	E_{9}	E_{13}	G_{9}	L_{9}	V_{10}	E_{9}	V_{9}	Q_{9}	G_{9}	S_{13}	S_{8}	E_{9}	E_{9}	S_{9}	F_{9}	V_{9}	L_{9}	R_{9}	W_{9}	C_{9}	A_{10}	F_{13}	A_{9}	T_{9}	L_{10}	F_{9}	L_{9}	S_{9}	F_{9}	I_{9}	N_{9}	Y_{9}
A_{4}	M_{4}	Q_{3}	F_{4}	E_{4}	E_{4}	G_{4}	L_{4}	V_{3}	L_{1}	V_{5}	Q_{4}	G_{4}	S_{4}	K_{4}	E_{4}	E_{4}	E_{1}	F_{4}	V_{4}	L_{4}	R_{4}	W_{4}	C_{4}	A_{4}	F_{4}	A_{5}	T_{4}	L_{4}	T_{1}	L_{5}	S_{4}	F_{4}	M_{3}	N_{4}	Y_{4}	D_{4}	W_{4}	A_{4}	V_{4}
S_{1}	Q_{1}	A_{1}	I_{1}	Q_{1}	Y_{1}	E_{1}	E_{1}	G_{1}			E_{1}		Q_{1}	G_{1}	S_{1}	K_{1}		K_{1}	S_{1}	F_{1}	V_{1}	L_{1}	R_{1}	W_{1}	C_{1}		F_{1}				F_{1}		I_{1}	F_{1}	I_{1}	N_{1}	Y_{1}	D_{1}	W_{1}
								I_{1}										E_{1}															S_{1}						

				685					690					695					700					705					710					715					720
D_{9}	W_{9}	A_{9}	V_{9}	G_{9}	S_{10}	V_{9}	S_{9}	A_{9}	I_{9}	G_{9}	K_{10}	M_{9}	K_{9}	T_{9}	V_{6}	F_{9}	G_{9}	A_{9}	L_{9}	G_{9}	P_{9}	D_{9}	F_{9}	I_{9}	E_{9}	S_{10}	Q_{9}	S_{12}	G_{9}	D_{13}	D_{10}	N_{9}	D_{9}	E_{9}	L_{9}	S_{6}	F_{9}	T_{9}	T_{9}
G_{4}	S_{4}	V_{4}	S_{5}	A_{4}	I_{4}	G_{4}	K_{4}	M_{4}	K_{5}	T_{4}	M_{4}	F_{4}	G_{4}	A_{4}	L_{4}	G_{4}	P_{4}	D_{4}	F_{4}	I_{4}	E_{4}	S_{4}	Q_{4}	S_{3}	G_{4}	D_{4}	E_{2}	D_{1}	D_{5}	N_{1}	L_{4}	K_{4}	F_{4}	T_{4}	T_{4}	F_{4}	E_{4}	V_{4}	E_{5}
A_{1}	V_{1}	G_{1}		V_{1}		A_{1}	I_{1}	G_{1}		M_{1}		T_{1}	I_{1}	F_{1}	I_{2}	A_{1}	L_{1}	G_{1}	P_{1}	D_{1}	F_{1}	I_{1}	E_{1}	N_{2}	Q_{1}		D_{2}	N_{1}				E_{1}	L_{1}	S_{1}	F_{1}	K_{3}	T_{1}	F_{1}	
															G_{1}												G_{1}									T_{1}			

725 730 735 740 745 750 755 760

$F_{9}\ E_{10}\ V_{9}\ E_{13}\ M_{9}\ P_{9}\ G_{9}\ E_{9}\ A_{9}\ G_{9}\ T_{9}\ S_{10}\ D_{9}\ S_{9}\ Q_{9}\ C_{9}\ F_{9}\ G_{9}\ D_{9}\ W_{9}\ L_{9}\ D_{9}\ H_{9}\ C_{9}\ I_{9}\ K_{9}\ Y_{9}\ N_{9}\ L_{9}\ T_{9}\ A_{9}\ I_{9}\ E_{9}\ P_{9}\ T_{9}\ T_{9}\ S_{9}\ G_{9}\ P_{9}\ M_{10}$

$M_{4}\ P_{4}\ G_{4}\ P_{1}\ A_{4}\ G_{4}\ T_{4}\ S_{4}\ D_{4}\ S_{5}\ Q_{4}\ C_{4}\ F_{4}\ G_{4}\ D_{4}\ W_{4}\ L_{4}\ E_{4}\ H_{4}\ C_{4}\ M_{3}\ K_{4}\ Y_{4}\ N_{4}\ L_{4}\ T_{4}\ A_{4}\ I_{4}\ E_{4}\ P_{4}\ T_{4}\ T_{4}\ S_{4}\ G_{4}\ P_{4}\ M_{4}\ L_{4}\ T_{4}\ L_{5}\ T_{4}$

$V_{1}\ M_{1}\ G_{1}\ E_{1}\ A_{1}\ G_{1}\ T_{1}\ D_{1}\ Q_{1}\ C_{1}\ F_{1}\ G_{1}\ D_{1}\ W_{1}\ L_{1}\ D_{1}\ H_{1}\ C_{1}\ I_{1}\ K_{1}\ Y_{1}\ N_{1}\ L_{1}\ T_{1}\ A_{1}\ I_{1}\ E_{1}\ P_{1}\ T_{1}\ T_{1}\ S_{1}\ G_{1}\ P_{1}\ M_{1}$

I_{1}

765 770 775 780 785 790 795 800

$L_{10}\ T_{9}\ L_{9}\ V_{9}\ K_{10}\ D_{9}\ K_{9}\ A_{13}\ N_{9}\ E_{9}\ L_{9}\ A_{9}\ D_{9}\ K_{9}\ M_{9}\ Q_{9}\ S_{9}\ L_{9}\ N_{10}\ A_{8}\ N_{9}\ D_{9}\ I_{8}\ R_{9}\ V_{12}\ H_{9}\ G_{9}\ G_{14}\ V_{9}\ G_{10}\ T_{13}\ G_{9}\ K_{9}\ S_{9}\ T_{9}\ A_{9}\ L_{13}\ P_{9}\ R_{9}\ E_{9}$

$K_{4}\ D_{3}\ S_{4}\ A_{4}\ G_{4}\ E_{4}\ L_{4}\ E_{1}\ D_{4}\ K_{4}\ M_{4}\ Q_{4}\ S_{4}\ L_{4}\ N_{4}\ A_{4}\ C_{4}\ D_{4}\ I_{3}\ R_{4}\ V_{4}\ H_{4}\ G_{4}\ G_{4}\ G_{1}\ G_{5}\ T_{4}\ K_{4}\ S_{4}\ K_{1}\ A_{4}\ L_{4}\ P_{4}\ S_{3}\ E_{4}\ R_{1}\ I_{3}\ S_{4}\ F_{4}$

$E_{1}\ K_{1}\ D_{1}\ A_{1}\ N_{1}\ L_{1}\ A_{1}\ D_{1}\ K_{1}\ M_{1}\ Q_{1}\ S_{1}\ L_{1}\ N_{1}\ A_{1}\ V_{1}\ D_{1}\ I_{1}\ R_{1}\ V_{2}\ H_{1}\ I_{1}\ V_{1}\ T_{1}\ S_{1}\ T_{1}\ A_{1}\ L_{1}\ P_{1}\ E_{1}\ L_{1}\ M_{1}$

$V_{1}\ T_{1}\ R_{1}\ M_{1}$

805 810 815 820 825 830 835 840

$L_{9}\ M_{9}\ R_{9}\ F_{8}\ G_{9}\ A_{9}\ V_{13}\ L_{9}\ M_{9}\ C_{9}\ V_{13}\ P_{9}\ T_{9}\ R_{9}\ V_{9}\ L_{13}\ A_{10}\ N_{9}\ A_{9}\ L_{9}\ H_{9}\ E_{9}\ S_{9}\ F_{9}\ M_{9}\ A_{9}\ L_{9}\ Y_{9}\ G_{9}\ F_{9}\ D_{9}\ V_{9}\ S_{9}\ L_{9}\ A_{9}\ Y_{9}\ R_{14}\ G_{9}\ R_{10}\ V_{9}$

$G_{4}\ S_{4}\ V_{4}\ L_{4}\ I_{4}\ C_{4}\ M_{1}\ P_{4}\ T_{4}\ R_{4}\ T_{1}\ L_{4}\ A_{4}\ N_{4}\ A_{5}\ N_{1}\ H_{4}\ E_{4}\ S_{4}\ Y_{4}\ M_{4}\ A_{4}\ L_{4}\ Y_{4}\ G_{4}\ F_{4}\ D_{4}\ V_{4}\ S_{4}\ L_{4}\ A_{4}\ Y_{4}\ R_{4}\ G_{4}\ R_{5}\ V_{4}\ T_{4}\ G_{4}\ S_{4}$

$R_{1}\ F_{1}\ G_{1}\ A_{1}\ V_{1}\ L_{1}\ C_{1}\ V_{1}\ P_{1}\ R_{1}\ V_{1}\ L_{1}\ L_{1}\ H_{1}\ E_{1}\ S_{1}\ F_{1}\ M_{1}\ A_{1}\ L_{1}\ Y_{1}\ G_{1}\ F_{1}\ D_{1}\ V_{1}\ S_{1}\ L_{1}\ A_{1}\ Y_{1}\ G_{1}\ V_{1}\ T_{1}$

Y_{1}

845 850 855 860 865 870 875 880

$R_{9}\ T_{9}\ G_{9}\ S_{8}\ K_{9}\ P_{9}\ I_{9}\ T_{9}\ V_{9}\ M_{9}\ T_{9}\ Y_{10}\ G_{9}\ Y_{9}\ A_{9}\ L_{9}\ N_{9}\ H_{10}\ F_{9}\ H_{9}\ H_{9}\ N_{9}\ P_{8}\ K_{9}\ N_{9}\ L_{9}\ A_{9}\ Q_{10}\ F_{10}\ Q_{9}\ F_{9}\ V_{9}\ L_{9}\ L_{9}\ D_{9}\ E_{9}\ V_{9}\ H_{9}\ T_{9}\ F_{9}$

$K_{4}\ P_{4}\ M_{4}\ T_{4}\ I_{5}\ M_{4}\ T_{4}\ Y_{4}\ G_{4}\ Y_{5}\ A_{4}\ L_{4}\ N_{4}\ H_{4}\ F_{4}\ H_{5}\ H_{4}\ N_{4}\ P_{4}\ K_{4}\ N_{4}\ L_{4}\ A_{4}\ Q_{4}\ F_{4}\ Q_{5}\ F_{5}\ V_{4}\ I_{2}\ L_{4}\ D_{4}\ E_{4}\ V_{4}\ H_{4}\ T_{4}\ F_{4}\ P_{4}\ V_{4}\ H_{4}\ L_{4}$

$G_{1}\ S_{1}\ K_{1}\ P_{1}\ T_{1}\ V_{1}\ M_{1}\ T_{1}\ G_{1}\ A_{1}\ L_{1}\ N_{1}\ F_{1}\ H_{1}\ N_{1}\ P_{1}\ K_{1}\ N_{1}\ L_{1}\ A_{1}\ V_{2}\ V_{1}\ L_{1}\ L_{1}\ D_{1}\ E_{1}\ V_{1}\ H_{1}\ T_{1}\ F_{1}\ P_{1}\ V_{1}$

885 890 895 900 905 910 915 920

$P_{9}\ V_{9}\ H_{9}\ L_{9}\ N_{9}\ P_{9}\ L_{9}\ F_{9}\ S_{9}\ L_{13}\ M_{9}\ S_{9}\ E_{13}\ L_{9}\ S_{9}\ P_{9}\ E_{9}\ K_{13}\ K_{9}\ I_{9}\ V_{9}\ K_{9}\ T_{9}\ S_{9}\ A_{9}\ T_{9}\ H_{9}\ V_{13}\ G_{9}\ Y_{9}\ N_{9}\ V_{9}\ D_{9}\ L_{9}\ S_{9}\ T_{9}\ N_{9}\ H_{9}\ K_{9}\ V_{9}$

$N_{4}\ P_{4}\ L_{4}\ F_{4}\ S_{4}\ L_{4}\ I_{4}\ R_{4}\ E_{4}\ S_{1}\ S_{4}\ P_{4}\ S_{1}\ K_{4}\ K_{4}\ I_{4}\ V_{4}\ I_{1}\ T_{4}\ S_{4}\ A_{4}\ T_{4}\ H_{4}\ V_{4}\ G_{4}\ H_{4}\ N_{4}\ H_{1}\ D_{4}\ L_{4}\ S_{4}\ T_{4}\ N_{4}\ H_{4}\ K_{4}\ V_{4}\ E_{4}\ I_{3}\ H_{4}\ T_{4}$

$H_{1}\ L_{1}\ N_{1}\ P_{1}\ L_{1}\ F_{1}\ S_{1}\ L_{1}\ M_{1}\ E_{1}\ L_{1}\ P_{1}\ E_{1}\ K_{1}\ K_{1}\ V_{1}\ K_{1}\ T_{1}\ S_{1}\ A_{1}\ T_{1}\ H_{1}\ V_{1}\ G_{1}\ N_{1}\ V_{1}\ D_{1}\ L_{1}\ S_{1}\ T_{1}\ N_{1}\ H_{1}\ K_{1}\ V_{1}\ D_{1}\ I_{1}$

M_{1}

925 930 935 940 945 950 955 960

$D_{9}\ I_{9}\ H_{9}\ T_{9}\ L_{10}\ G_{9}\ L_{8}\ M_{9}\ D_{9}\ V_{9}\ K_{9}\ K_{9}\ W_{9}\ A_{9}\ E_{9}\ M_{9}\ Q_{9}\ G_{10}\ T_{9}\ G_{9}\ V_{9}\ F_{9}\ G_{9}\ D_{13}\ T_{9}\ T_{9}\ K_{9}\ D_{9}\ T_{9}\ G_{9}\ N_{9}\ V_{14}\ L_{9}\ V_{10}\ F_{9}\ V_{9}\ A_{9}\ S_{9}\ Y_{9}\ K_{9}$

$L_{4}\ G_{4}\ L_{5}\ M_{4}\ D_{4}\ V_{4}\ K_{4}\ K_{4}\ W_{4}\ A_{4}\ E_{4}\ M_{4}\ Q_{4}\ G_{4}\ T_{4}\ G_{5}\ V_{4}\ F_{4}\ G_{4}\ D_{4}\ A_{3}\ T_{4}\ K_{3}\ T_{1}\ S_{4}\ G_{4}\ N_{4}\ V_{4}\ L_{4}\ V_{5}\ F_{4}\ A_{4}\ S_{4}\ Y_{4}\ K_{4}\ D_{4}\ V_{4}\ D_{5}\ A_{4}$

$H_{1}\ T_{1}\ G_{1}\ M_{1}\ D_{1}\ V_{1}\ K_{1}\ K_{1}\ W_{1}\ A_{1}\ E_{1}\ M_{1}\ Q_{1}\ T_{1}\ V_{1}\ F_{1}\ G_{1}\ D_{1}\ S_{1}\ K_{1}\ D_{1}\ T_{1}\ G_{1}\ N_{1}\ L_{1}\ F_{1}\ A_{1}\ S_{1}\ Y_{1}\ K_{1}\ V_{1}$

$T_{1}\ T_{1}$

965 970 975 980 985 990 995 1000

$D_{10}\ V_{9}\ D_{9}\ T_{9}\ C_{9}\ A_{9}\ E_{9}\ K_{13}\ L_{9}\ S_{9}\ D_{9}\ K_{9}\ G_{9}\ L_{9}\ P_{9}\ V_{8}\ L_{9}\ K_{9}\ V_{9}\ D_{9}\ G_{9}\ S_{8}\ N_{13}\ F_{9}\ R_{9}\ K_{8}\ N_{9}\ T_{9}\ D_{9}\ V_{13}\ Q_{10}\ R_{9}\ Q_{9}\ V_{11}\ D_{9}\ E_{11}\ L_{8}\ V_{9}\ G_{9}\ D_{9}$

$C_{4}\ A_{4}\ E_{4}\ K_{4}\ L_{4}\ S_{4}\ D_{4}\ S_{1}\ G_{4}\ L_{4}\ P_{4}\ V_{4}\ L_{4}\ K_{4}\ V_{4}\ D_{4}\ G_{4}\ S_{4}\ N_{4}\ F_{4}\ R_{4}\ K_{4}\ R_{1}\ T_{4}\ D_{4}\ V_{4}\ Q_{4}\ R_{4}\ Q_{5}\ R_{1}\ D_{4}\ E_{4}\ M_{4}\ I_{1}\ G_{4}\ D_{2}\ T_{3}\ K_{4}\ F_{4}\ I_{4}$

$T_{1}\ C_{1}\ A_{1}\ E_{1}\ K_{1}\ L_{1}\ D_{1}\ K_{1}\ G_{1}\ L_{1}\ P_{1}\ V_{1}\ L_{1}\ I_{1}\ V_{1}\ D_{1}\ G_{1}\ S_{1}\ N_{1}\ F_{1}\ K_{1}\ N_{1}\ T_{1}\ D_{1}\ V_{1}\ V_{1}\ D_{1}\ E_{1}\ L_{1}\ V_{1}\ M_{2}\ D_{1}\ T_{1}\ K_{1}$

$K_{1}\ R_{1}\ S_{1}\ A_{1}\ G_{1}$

1005 1010 1015 1020 1025 1030 1035 1040

$T_{9}\ K_{9}\ F_{9}\ I_{8}\ I_{6}\ A_{9}\ T_{9}\ N_{13}\ M_{9}\ M_{8}\ E_{9}\ N_{9}\ G_{9}\ V_{13}\ T_{9}\ L_{9}\ D_{10}\ V_{14}\ D_{13}\ V_{10}\ V_{9}\ V_{9}\ D_{9}\ F_{9}\ G_{9}\ E_{9}\ K_{9}\ V_{9}\ S_{9}\ P_{9}\ G_{8}\ L_{9}\ F_{9}\ S_{9}\ E_{9}\ E_{9}\ S_{9}\ C_{9}\ V_{9}\ L_{9}$

$V_{4}\ A_{4}\ T_{4}\ N_{4}\ M_{4}\ V_{4}\ E_{4}\ M_{1}\ G_{4}\ V_{4}\ T_{4}\ L_{4}\ D_{4}\ L_{1}\ D_{5}\ V_{5}\ V_{4}\ V_{1}\ F_{4}\ G_{4}\ E_{4}\ K_{4}\ V_{4}\ S_{4}\ P_{4}\ E_{3}\ L_{4}\ F_{4}\ S_{4}\ E_{4}\ E_{4}\ S_{4}\ C_{4}\ V_{4}\ L_{4}\ L_{4}\ R_{4}\ R_{4}\ Q_{4}$

$F_{1}\ I_{1}\ V_{1}\ M_{1}\ V_{3}\ N_{1}\ M_{1}\ E_{1}\ I_{1}\ G_{1}\ V_{1}\ T_{1}\ D_{1}\ F_{1}\ G_{1}\ E_{1}\ K_{1}\ V_{1}\ S_{1}\ P_{1}\ G_{1}\ L_{1}\ F_{1}\ S_{1}\ E_{1}\ E_{1}\ S_{1}\ C_{1}\ V_{1}\ L_{1}\ L_{1}\ H_{1}$

$A_{1}\ T_{1}\ N_{1}\ D_{1}\ S_{1}$

1045 1050 1055 1060 1065 1070 1075 1080

$L_{9}\ H_{9}\ S_{13}\ Q_{13}\ R_{9}\ I_{9}\ S_{13}\ Q_{9}\ A_{9}\ E_{9}\ S_{9}\ K_{9}\ Q_{9}\ R_{7}\ F_{9}\ G_{9}\ S_{8}\ V_{9}\ G_{9}\ S_{9}\ M_{9}\ K_{9}\ K_{9}\ G_{9}\ T_{9}\ V_{9}\ Y_{9}\ K_{9}\ F_{9}\ G_{9}\ R_{9}\ E_{9}\ T_{9}\ L_{9}\ P_{9}\ D_{9}\ S_{10}\ M_{13}\ S_{9}\ N_{9}$

$S_{5}\ I_{4}\ R_{1}\ I_{1}\ A_{4}\ E_{4}\ A_{1}\ K_{4}\ Q_{4}\ S_{4}\ F_{4}\ G_{4}\ S_{4}\ V_{4}\ G_{4}\ S_{4}\ M_{4}\ K_{4}\ K_{4}\ G_{4}\ T_{3}\ I_{3}\ Y_{4}\ K_{4}\ F_{4}\ G_{4}\ K_{4}\ E_{4}\ T_{4}\ L_{4}\ P_{4}\ D_{4}\ S_{4}\ M_{4}\ S_{5}\ N_{4}\ R_{4}\ N_{1}\ G_{4}\ S_{4}$

$Q_{1}\ S_{1}\ Q_{1}\ E_{1}\ S_{1}\ K_{1}\ Q_{1}\ R_{1}\ F_{1}\ S_{2}\ S_{1}\ V_{1}\ G_{1}\ S_{1}\ M_{1}\ K_{1}\ K_{1}\ G_{1}\ T_{1}\ V_{1}\ Y_{1}\ K_{1}\ F_{1}\ G_{1}\ R_{1}\ E_{1}\ T_{1}\ L_{1}\ P_{1}\ D_{1}\ M_{1}\ R_{1}\ M_{1}$

$G_{1}\ R_{1}\ V_{1}\ M_{1}$

1085 1090 1095 1100 1105 1110 1115 1120

$R_{9}\ M_{9}\ G_{9}\ S_{9}\ T_{9}\ E_{9}\ S_{9}\ A_{9}\ L_{9}\ L_{9}\ C_{9}\ F_{9}\ A_{9}\ Y_{9}\ G_{9}\ L_{9}\ K_{9}\ P_{9}\ V_{13}\ V_{9}\ D_{9}\ D_{10}\ V_{10}\ D_{9}\ V_{9}\ S_{9}\ A_{9}\ V_{9}\ S_{13}\ K_{13}\ I_{9}\ T_{9}\ S_{9}\ K_{9}\ Q_{9}\ A_{9}\ L_{9}\ T_{9}\ A_{9}\ S_{9}$

$T_{4}\ E_{4}\ S_{4}\ A_{4}\ L_{4}\ L_{4}\ C_{4}\ F_{4}\ A_{4}\ Y_{4}\ G_{4}\ L_{4}\ K_{4}\ P_{4}\ V_{4}\ V_{4}\ D_{4}\ D_{4}\ D_{1}\ D_{5}\ I_{3}\ S_{4}\ A_{4}\ V_{4}\ S_{3}\ K_{4}\ I_{4}\ T_{4}\ I_{1}\ T_{1}\ Q_{4}\ A_{4}\ L_{4}\ T_{4}\ A_{4}\ S_{4}\ M_{4}\ F_{4}\ E_{4}\ A_{4}$

$G_{1}\ S_{1}\ T_{1}\ E_{1}\ S_{1}\ A_{1}\ L_{1}\ L_{1}\ C_{1}\ F_{1}\ A_{1}\ Y_{1}\ G_{1}\ L_{1}\ K_{1}\ P_{1}\ V_{1}\ V_{1}\ V_{2}\ S_{1}\ K_{1}\ V_{1}\ K_{1}\ K_{1}\ S_{1}\ K_{1}\ Q_{1}\ A_{1}\ L_{1}\ T_{1}\ A_{1}\ S_{1}\ M_{1}\ F_{1}$

A_{1}

1125 1130 1135 1140 1145 1150 1155 1160

$M_{9}\ F_{9}\ E_{9}\ A_{9}\ N_{9}\ Y_{9}\ M_{9}\ F_{8}\ T_{9}\ A_{8}\ H_{9}\ L_{9}\ V_{9}\ D_{9}\ S_{9}\ Q_{9}\ G_{9}\ F_{9}\ M_{9}\ P_{9}\ S_{9}\ P_{9}\ V_{9}\ F_{9}\ E_{9}\ L_{13}\ M_{9}\ K_{9}\ N_{9}\ L_{10}\ L_{9}\ L_{9}\ H_{9}\ T_{9}\ D_{9}\ A_{9}\ V_{9}\ G_{9}\ I_{9}\ C_{9}$

$N_{4}\ Y_{4}\ M_{4}\ F_{4}\ T_{4}\ A_{4}\ H_{4}\ L_{4}\ V_{4}\ D_{4}\ S_{4}\ Q_{4}\ G_{4}\ F_{4}\ M_{4}\ P_{4}\ R_{4}\ P_{5}\ V_{4}\ F_{4}\ E_{4}\ L_{4}\ M_{4}\ K_{4}\ N_{4}\ K_{1}\ L_{4}\ L_{5}\ H_{4}\ T_{4}\ D_{4}\ A_{4}\ V_{4}\ G_{4}\ M_{4}\ C_{4}\ S_{4}\ T_{4}\ Y_{4}\ L_{4}$

$E_{1}\ A_{1}\ N_{1}\ Y_{1}\ M_{1}\ F_{1}\ T_{1}\ A_{1}\ H_{1}\ L_{1}\ V_{1}\ D_{1}\ S_{1}\ Q_{1}\ G_{1}\ F_{1}\ M_{1}\ S_{1}\ V_{1}\ F_{1}\ E_{1}\ L_{1}\ M_{1}\ N_{1}\ L_{1}\ H_{1}\ T_{1}\ D_{1}\ A_{1}\ V_{1}\ G_{1}\ I_{1}\ C_{1}\ S_{1}\ S_{1}$

1165 1170 1175 1180 1185 1190 1195 1200

S8 S9 Y9 L9 A9 T9 N9 M9 S9 E9 W9 S9 R8 V9 C9 E9 Y9 I9 K9 I8 D9 E9 S9 S9 R9 H9 V9 Q9 E9 V9 K8 I9 P9 W9 Y9 C9 S9 D13 M9 S9
A4 T4 N4 M4 S4 E4 W4 S4 R4 V4 C4 E4 Y4 I4 K4 I3 D3 M5 S4 S4 S5 H4 V3 Q4 E4 V4 K4 M4 P4 W4 Y4 C4 S4 D4 M4 S4 D4 S1 F4 I4
Y1 L1 A1 T1 N1 M1 S1 E1 W1 S1 R1 V1 C1 E1 Y1 T2 K1 D3 D1 E1 S1 M1 H1 V1 Q1 E1 V1 K1 I1 P1 W1 Y1 C1 S1 D1 M1 D1 D1
N1 E1 E1 V1 R1 S1

1205 1210 1215 1220 1225 1230 1235 1240

D9 D9 F9 I9 V9 K9 L9 A9 E9 C9 V9 K9 A9 A9 K10 P9 K9 L8 S12 S9 G9 Y9 K9 V9 D9 N9 V9 D9 F9 H13 T9 V9 A9 H9 K9 M9 S13 V9 G9 E9
V4 K4 L4 A4 E4 C4 V4 K4 A4 S4 K4 P4 K5 L4 S4 S4 G4 Y4 G1 V4 D4 N4 V4 D4 F4 H4 T4 V4 A4 V1 K4 M4 S4 V4 G4 E4 G1 N4 I4 D4
F1 I1 V1 K1 L1 A1 E1 C1 V1 K1 A1 A1 P1 L1 S1 F1 K1 Y1 K1 V1 D1 N1 V1 D1 F1 H1 T1 A1 H1 K1 M1 S1 V1 E1 S1 N1
S1

1245 1250 1255 1260 1265 1270 1275 1280

S9 N9 I9 D9 E9 S9 S9 A9 L9 V9 A9 T9 I9 L9 D9 E9 V9 K9 Q9 W9 S9 D9 G9 M9 T9 Y9 H9 S9 S9 T9 P9 S9 N9 K9 S9 L9 M9 S9 L9 M9
E4 S4 S4 S5 L4 V4 A4 T4 I4 L4 D4 E4 V4 K4 Q4 W4 S4 D4 G4 M4 T4 Y4 H4 S4 S4 T4 P4 R4 N4 K3 S4 L4 M4 S4 L4 M4 V4 G4 W4 I4
I1 D1 E1 S1 A1 L1 V1 A1 T1 I1 L1 D1 E1 V1 K1 Q1 W1 S1 D1 G1 M1 T1 Y1 H1 S1 S1 T1 P1 S2 N1 K1 S1 L1 M1 S1 L1 M1 V1 G1

1285 1290 1295 1300 1305 1310 1315 1320

V9 G9 W9 I9 P9 R9 K9 A9 E9 S9 T9 K9 A9 M9 L9 D9 E9 R9 V9 Q9 S9 L14 E9 L10 L13 L9 N9 Q9 L9 N9 G9 V9 K9 G9 V9 D9 D9 Y9 E9 S9
P4 R4 K4 A4 E4 K4 T4 K4 A4 I4 L4 D4 E4 R4 V4 Q4 R4 L4 E4 L5 L4 N4 Q4 N1 N4 G4 V4 S4 G4 I4 D4 D4 Y4 E4 S4 L4 V4 S4 F4
W1 I1 P1 R1 K1 A1 E1 S1 T1 K1 A1 M1 L1 D1 E1 R1 V1 Q1 S1 E1 L1 Q1 L1 N1 G1 V1 K1 G1 V1 D1 D1 Y1 E1 S1 L1 V1

1325 1330 1335 1340 1345 1350 1355 1360

L9 V9 S9 F9 F9 S9 E9 N9 P9 H9 S9 A9 E9 Y9 L9 E9 A9 Q9 C9 A9 S9 D9 Y9 I9 E9 E9 K9 V9 M9 N13 V9 K9 K13 N9 Y9 D9 K9 P8 I10 I9
F4 S4 E4 N4 P4 H4 S4 A4 E4 Y4 L4 E4 A4 Q4 C4 A4 S4 D4 Y4 I4 E4 E4 K4 V4 M4 N2 V4 K4 R3 K1 Y4 D4 Y1 P4 M4 I4 I4 G4 L4 V4
S1 F1 F1 S1 E1 N1 P1 H1 S1 A1 E1 Y1 L1 E1 A1 Q1 C1 A1 S1 D1 Y1 I1 E1 E1 K1 S2 M1 N1 S1 K1 N1 D1 K1 P1 V1 I1 G1
V1 V1 S1

1365 1370 1375 1380 1385 1390 1395 1400

I9 G9 L9 V13 G9 L9 A6 V9 A5 T10 G9 T9 F9 A9 Y10 W9 Y9 M6 S9 S13 N9 A9 A9 S9 E9 V9 V8 E8 S9 Q9 A9 K10 H9 K9 Y8 N9 R9 D9 K9 R9
G4 L4 A4 L1 A5 T4 G4 T5 F4 A4 Y4 W4 Y5 M3 R4 S4 E4 A4 A4 A1 E4 V4 V4 E4 K4 Q4 A4 K4 H4 K5 Y4 N4 R4 D4 K4 S4 T4 G4 S4 L4
L1 V1 G1 V1 S3 T4 F1 A1 L1 M1 S1 L3 N1 A1 S1 E1 V1 V1 E1 I1 G1 A1 H1 Y1 N1 F1 D1 K1 R1 T1 G1
T1 G1 W1 S1 S1 Q1 R1

1405 1410 1415 1420 1425 1430 1435 1440

T9 G9 S9 L9 M9 Y9 D10 L9 D9 D10 Q9 G6 T9 V9 E9 T9 F9 G9 V9 E9 Y9 S9 D9 A9 V9 M12 T9 G9 K9 M9 S9 K9 A9 Q9 K9 E10 R9 E9 S9 R9
M4 F3 D4 Y5 S3 D4 Q4 D5 T4 V4 E4 T4 F4 G4 V4 E4 Y4 S4 D4 A4 V4 I4 T4 G4 K4 G1 S4 K4 A4 Q4 K4 E4 R4 E5 S4 R4 K4 K4 G4 W4
S1 L1 M1 D1 L1 Q1 T1 S3 E1 T1 F1 G1 V1 E1 Y1 S1 D1 A1 V1 M1 T1 I1 K1 M1 S1 K1 A1 Q1 K1 R1 S1 R1 K1 K1
Y1 N1 V1

1445 1450 1455 1460 1465 1470 1475 1480

K13 K9 G13 W9 K9 V9 G9 K9 V9 N9 R9 P9 M9 R9 V9 F9 R9 Q9 L9 Y9 G9 V9 N9 P9 L9 E9 F9 D9 E9 V13 V9 M9 R9 V9 G9 E9 L9 T9 S10 E9
G1 I4 K1 K4 V4 N4 R4 P4 M4 R4 V4 F4 H4 Q4 L4 Y4 G4 V4 N4 P4 L4 E4 F4 D4 E4 V4 V3 M4 R4 M1 G4 D4 W4 A4 T4 D4 P4 W4 T3 A4
W1 V1 G1 K1 V1 D1 R1 P1 M1 R1 V1 F1 R1 Q1 L1 Y1 G1 V1 N1 P1 L1 E1 F1 D1 I1 V1 V1 R1 V1 G1 E1 L1 T1 S1 E1 P1 W1
E1

1485 1490 1495 1500 1505 1510 1515 1520

P9 W9 S9 A9 K9 D14 V9 D10 I9 D9 N9 M9 L9 V7 E9 L9 D10 D9 D9 F7 H9 I9 L9 S8 G9 D9 S8 M9 L9 G9 K9 K9 V9 E9 L9 A9 F9 T9 K9 D12
K4 D3 V4 N4 V4 G4 M4 L4 I1 E4 L3 D4 D3 D4 Y4 H4 I3 L3 K4 D4 D4 R4 M4 L3 G3 K5 K1 V4 E4 L3 A4 F4 T4 K3 S3 G4 S4 G2 S1
S1 A1 K1 D1 I1 N1 M1 L1 V1 E1 I2 D1 Y1 H1 Y2 L1 S1 G1 K1 F1 M1 L1 G1 K1 K1 V1 E1 L1 A1 F1 T1 K1 D1 S2 N1
L1 I1 D1 S1 E1 N1
S1

1525 1530 1535 1540 1545 1550 1555 1560

G9 S9 N9 E9 E9 T9 V9 V9 K9 L9 T9 P9 H9 R9 S13 K9 M13 A9 S9 S13 M9 S10 L9 N5 P13 M9 G9 F9 P9 E10 E9 E9 G9 R9 W9 S12 Q9 T9 G9 S9
E4 T4 V4 V3 K2 L4 T3 P4 H4 R4 S4 S3 M4 A4 M1 S4 S1 S5 L4 P4 M4 G3 F4 G1 E4 E4 E5 G4 R4 W4 S4 Q4 T4 G4 N1 P4 V4 V4 Q3
S1 E1 E1 T1 Q1 V1 K1 L1 T1 P1 H1 R1 S1 K1 A1 M1 L1 P1 S4 F1 P1 E1 G1 R1 W1 S1 Q1 T1 G1 S1 P1 R1
V1 M1 V1

				1565					1570					1575					1580					1585					1590					1595					1600
P9	V6	V9	Q9	K7	S8	S8	E8	E9	N7	Q9	S9	V12	E9	K13	Q9	V9	A9	K9	P9	E9	T9	T9	N9	P9	Y9	E9	H9	V10	L9	V9	R9	L9	G9	S9	A9	H9	L9	G9	T8
K4	S3	F2	E4	D3	G3	Q3	T4	V4	E4	M3	Q4	I1	A4	V1	P4	E4	T2	T4	N4	P4	Y4	E4	H4	V4	L4	V5	R4	L4	G4	S4	A4	H4	L4	G4	T4	S4	V4	L4	N4
V1	M2	K1	S1	S2	K1	E1	K1	Q1	S2	T1	E1	K1	Q1		A1	K1	V2	E1	T1	T1	N1	P1	Y1	E1	H1		L1		R1	L1	G1	S1	A1	H1	L1	G1	T1	S1	S1
	K1	T1		E2	D1	H1	N1		D1	V1							P1																						V1
	L1	S1			E1	L1																																	
	Q1																																						

				1605					1610					1615					1620					1625					1630					1635					1640
S9	V9	L9	N9	C9	F9	F9	H9	G9	S9	K9	C9	V9	M9	P9	Y9	H9	L9	A9	E9	N10	G9	D9	A9	N9	E9	S13	L9	V9	M9	S9	T9	T9	R9	G9	Q9	F10	D9	F9	G9
C4	F4	F4	H4	G4	S4	K4	C4	V4	I4	P4	Y4	H4	L4	A4	E4	N4	G4	D4	L4	D3	K2	S3	L4	I3	I4	V1	T4	T4	R4	G4	Q4	F4	D4	F5	G4	P4	L4	K4	N4
L1	N1	C1	F1	F1	H1	G1	S1	K1	C1	V1	M1	P1	Y1	H1	L1	A1	E1	N1	G1	S1	E2	N1	E1	S1	L1		M1	S1	T1	T1	R1	G1	Q1		D1		G1	P1	L1
																					A1	P1		V1															

				1645					1650					1655					1660					1665					1670					1675					1680
P9	L9	K9	N9	I9	K10	C9	K9	K10	M9	K9	D10	Y9	D9	M9	T9	V9	C9	P10	L9	P9	R9	D9	V9	Q9	P9	F9	R9	A9	K9	I9	V9	F9	R9	E9	P9	K9	L9	G9	E9
I4	K4	C4	K5	K4	I4	K5	D4	Y4	D5	I4	T4	V3	C4	P4	L4	P5	S4	D4	V4	Q4	P4	F4	S4	A4	K4	M4	V4	F3	S4	E4	P4	K4	L4	G4	E4	E4	V4	V4	V5
K1	N1	I1		C1			M1			Y1		I1	T1	V1	C1		L1		R1	D1	V1	Q1	P1	F1	R1	A1	K1	I1	V1	F1	R1	E1	P1	K1	L1	G1	E1	E1	
												M1																V1											

				1685					1690					1695					1700					1705					1710					1715					1720
E9	V10	V10	V7	V9	Y9	F9	A9	S12	I13	D6	G9	S7	I9	V8	M9	K12	V7	S9	E9	K9	S13	N9	T9	Y9	Q9	A9	G9	G9	S9	F9	T12	H9	L9	W9	T9	Y9	Q9	H7	D9
V5	C2	F4	A4	S4	I4	D4	G4	R1	G1	V4	M4	K6	V3	S4	E4	S2	S4	N4	T4	Y4	T1	A4	G4	G4	L3	F4	T4	H4	L4	W4	A1	Y4	Q4	Y3	D4	G4	N4	P4	G4
	F1		I2	F1	A1	S1	I1	N1		N3	I1	V1	M1	I1	A1		A2	K1	S1	N1		Y1	Q1	A1	G1	G1	S1	F1	K1	H1	L1	W1	T1	H2	Q1	H1	D1	F2	N1
	Y1		Y1							S1			A1	K1			E1								S1													G1	

				1725					1730					1735					1740					1745					1750					1755					1760
G9	N9	P9	G13	D9	C9	G9	G9	P9	I9	V9	A9	T9	S9	D9	M9	K9	V9	V9	G13	F9	H9	S13	G9	V9	V9	S9	N9	G10	A9	G9	E9	K9	L9	R9	A9	V13	Y9	T9	P9
D4	C4	G4	C1	P4	I4	V4	A4	T4	S4	D4	L4	K4	V4	V3	G4	F4	H4	S4	H1	V4	V3	V1	N4	G3	A3	G5	E4	K4	L4	R4	A4	V4	Y4	T4	P4	T1	N4	Q4	E4
P1	G1	D1		G1	G1	P1	I1	V1	A1	T1	S1	D1	M1	I1	V1	V1	G1	F1		S1	G1		V1	S2	E1		A1		E1	K1	L1	R1	A1	V1	Y1		P1	V1	N1
														K1							A1				N1														

				1765					1770					1775					1780					1785					1790					1795					1800
V9	N8	H9	E9	L13	I9	A7	S10	L9	S9	T9	E9	I9	Q9	M9	T13	D9	F9	W9	T9	F9	N9	P9	D8	L9	V9	E9	W9	N9	S9	V9	A9	R9	V9	S9	T9	F5	F9	P9	M10
L4	I4	S4	S4	V1	S5	S4	E4	V4	Q4	M4	N2	D4	F4	W4	F1	F4	N4	P4	D2	L4	V4	E4	W4	N4	S4	V4	A4	R4	V4	S4	T4	F4	F4	P4	M4	Y4	K4	A3	I3
H1	E1	L1	I1			V2		T1	D1	V1	T1	M1	T1	D1		W1	T1	F1	K1	P1	D1	L1	N1	E1	W1	N1	S1	V1	A1	R1	V1	S1	T1	F1	F1	S4	M1	T1	K1
	K1					L1					Q1								N1				V1													P1		S1	
											D1								A1																				

				1805					1810					1815					1820					1825					1830					1835					1840
S9	K9	A9	M9	N9	A8	I9	T9	V10	Q9	V9	N8	D9	G9	E9	E9	S7	I9	D9	G9	N9	L9	M9	M9	V9	G9	S8	V13	N10	S9	N13	V9	Y9	H10	N9	H9	V9	M9	K11	G9
N4	T4	M4	T4	V4	Q4	V5	D3	D4	G4	E4	E4	M4	I4	D4	G4	N4	L4	M4	M4	V4	G4	S4	V4	N4	S4	T5	S1	Y4	H4	Y1	H5	V4	M4	K4	G4	K5	S4	E3	S5
A1	M1	N1	A1	I1	S2		Q1		N1	D1	G1	E1	E1	L1	I1	L2	G1	N1	L1	M1	M1	V1	G1	S1	V1	N1			V1			N1		V1	M1		G1		
							N1				D1					D1																							

				1845					1850					1855					1860					1865					1870					1875					1880
K9	S10	E8	S9	F9	V9	R8	Y9	C9	E9	Q9	F9	P9	K9	S9	A9	F9	T9	P9	E9	L9	S9	D8	Q9	Y9	L13	P9	S9	I9	L9	S9	K9	P9	A9	F9	S9	K13	G9	L9	L9
F4	V4	R4	Y4	C4	E3	Q5	F4	P4	H3	S4	A4	F4	V4	P4	E4	L4	S4	D2	K3	Y4	L4	P4	S4	I3	S1	S4	K4	P4	A4	F4	R4	K4	G4	L4	L4	L1	Y4	N4	E4
E1		F1	V1	R1	Y1	C1	E1	Q1	F1	P1	K1	S1	A1	F1	M1	P1	E1	N2	S2	D1	Q1	G1	L1	P1		I1	L1	S1	K1	P1	A1	F1	S1	K1	G1		L1	K1	Y1
		Q1			N1				N1									L1				Y1		V1															

				1885					1890					1895					1900					1905					1910					1915					1920
K9	Y9	N9	E9	P9	V10	R9	V9	G9	L7	V9	N9	F9	E9	C9	L9	V7	K5	A9	Y9	L9	V9	V9	E13	K9	K9	L9	E9	E9	L9	G8	F9	M9	G9	N9	S9	G9	P13	Q9	W9
P4	V4	K4	V5	G4	F4	V4	N4	Y4	E4	C4	L4	I4	K2	A4	Y4	L4	V4	I4	E4	K3	K4	L4	K1	E3	L4	E4	F4	R2	G4	N4	S4	G4	P4	E4	W4	D4	W1	M4	E4
N1	E1	P1		R1		G1	L1	V1	S2	F1	E1	C1	S2	V1	K1	M2	S4	L1	V1	S1	E1	K1		D1	E1	G1	L1	H1	F1	E1	G1	N1	S1	G1	P1	Q1		D1	P1
									N1				L1			A1	Y1			V1				L1				G1		V1									
																												C1											

				1925					1930					1935					1940					1945					1950					1955					1960
D_9	P_9	M_9	E_9	I_9	L_9	S_5	D_9	L_9	N_9	K_9	K_9	A_{13}	A_9	M_9	G_9	A_9	L_9	Y_9	Q_{13}	G_9	S_9	K_9	Q_9	D_9	W_9	L_9	K_9	S_9	M_9	T_9	P_9	E_9	E_9	F_9	I_9	M_5	S_{13}	V_9	R_9
I_4	L_4	S_3	D_4	L_4	N_4	K_4	K_4	A_4	A_4	M_4	G_4	M_1	L_4	Y_4	Q_4	G_4	S_4	K_4	S_1	D_4	W_4	L_4	K_4	S_4	M_4	S_5	P_4	E_4	D_4	F_4	V_3	V_4	S_3	V_5	R_4	V_5	R_1	F_4	K_4
M_1	E_1	I_1	L_1	N_1	D_1	N_3	N_1	K_1	K_1	A_1	A_1		G_1	A_1	L_1	Y_1	Q_1	G_1		K_1	Q_1	D_1	W_1	L_1	K_1		M_1	T_1	P_1	E_1	I_1	F_1	I_1		S_1	E_4		E_1	S_1
		N_1				D_1																									E_1		A_1						
						L_1																																	

				1965					1970					1975					1980					1985					1990					1995					2000
E_9	S_9	F_9	K_9	H_9	L_9	A_9	G_{13}	G_9	D_9	V_9	G_{13}	I_9	W_9	S_{10}	G_9	S_9	L_{13}	K_9	A_9	E_9	L_9	R_9	P_9	V_9	E_{13}	K_9	V_9	S_9	E_9	M_9	K_9	T_{13}	R_9	V_9	F_9	T_9	G_9	A_9	P_9
H_4	L_4	A_4	G_4	G_4	D_4	V_3	D_1	I_4	W_4	S_4	W_1	S_5	L_4	K_4	A_4	E_4	A_1	S_4	P_3	C_2	E_4	K_4	V_4	K_5	V_1	M_4	K_4	T_4	S_4	V_4	F_4	V_1	G_4	A_4	P_4	V_4	D_4	V_5	L_4
F_1	K_1	H_1	L_1	A_1	G_1	G_1		V_1	G_1	I_1			G_1		L_1	K_1		E_1	L_1	R_2	P_1	V_1	E_1			S_1	D_1	M_1	K_1	T_1	R_1		F_1	T_1	G_1	A_1	P_1		D_1
						I_1													T_1	Y_1																			

				2005					2010					2015					2020					2025					2030					2035					2040
V_{10}	D_9	V_9	L_9	L_9	G_9	G_9	K_9	V_{10}	L_9	V_9	D_9	N_{10}	F_{13}	N_9	H_8	H_9	F_9	Y_9	E_9	N_9	H_9	L_9	K_9	G_9	P_9	W_9	T_9	V_9	G_9	M_9	N_9	K_9	F_9	N_{10}	S_{10}	G_9	W_9	D_9	S_9
L_3	G_4	G_4	K_4	I_4	L_4	V_5	D_4	N_4	F_4	N_5	H_4	H_3	H_1	Y_4	E_4	N_4	H_4	L_4	K_4	G_4	P_4	W_4	T_4	V_4	G_4	I_4	N_4	K_4	F_4	N_4	R_4	G_4	W_4	D_3	K_3	L_4	A_4	S_3	Y_4
	L_1	L_1	G_1	G_1	K_1		L_1		D_1		F_1	Y_1		H_1	F_1	Y_1	E_1	N_1	H_1	L_1	K_1	G_1	P_1	W_1	T_1	V_1	G_1	M_1	N_1	K_1	F_1	N_1	S_1	G_1	W_1	D_1	S_1	E_1	A_1
																																						L_1	

				2045					2050					2055					2060					2065					2070					2075					2080
L_9	A_9	S_9	Y_9	F_9	N_{13}	H_9	G_9	W_9	N_9	F_9	I_9	D_{10}	C_9	D_9	G_9	S_9	R_9	F_9	D_9	T_9	S_9	L_{13}	A_9	P_9	M_9	L_9	F_9	Q_9	L_9	I_7	C_9	H_9	M_9	S_{10}	E_9	S_9	F_9	G_9	E_8
F_4	D_2	H_4	S_4	W_4	D_1	F_4	I_3	D_4	C_4	D_5	G_4	S_4	R_4	F_4	D_4	T_4	S_4	L_4	A_4	P_4	M_4	P_1	F_4	Q_4	L_4	V_4	C_4	H_4	M_3	S_4	E_4	S_5	F_4	G_4	G_4	F_4	D_4	A_4	M_4
S_1	N_2	F_1	N_1	H_1		W_1	N_1	F_1	I_1		C_1		G_1	S_1	R_1	F_1	D_1	T_1	S_1	L_1	A_1		M_1	L_1	F_1	Q_1	L_1	I_1	C_1	V_2	M_1		E_1		F_1	G_1	E_1	F_1	G_1
	Y_1						M_1																						V_1	H_1									D_1

				2085					2090					2095					2100					2105					2110					2115					2120
F_9	D_9	N_5	I_8	E_9	T_9	I_6	A_9	L_8	S_9	N_9	L_9	Y_9	T_9	Q_9	I_9	V_9	Y_9	T_{13}	P_9	I_9	L_9	T_9	I_{13}	D_9	G_9	Y_9	M_9	T_8	K_9	K_9	H_9	S_{13}	G_{13}	N_9	N_9	S_{13}	G_9	Q_9	P_9
E_4	T_4	S_5	A_4	L_4	S_4	N_4	L_4	Y_4	T_4	Q_4	I_4	V_4	Y_4	T_4	P_4	I_4	L_4	I_1	I_4	D_4	G_4	Y_4	G_1	T_3	K_4	K_4	H_4	S_4	G_4	N_4	N_4	N_1	N_1	Q_4	P_4	Q_1	T_4	V_4	V_4
N_1	I_1	A_3	V_1	I_1	A_1	V_2	S_1	M_1	L_1	Y_1	T_1	Q_1	I_1	V_1	Y_1	T_1	P_1		L_1	T_1	I_1	D_1		F_1	M_1	T_1	K_1	K_1	H_1	S_1	G_1			S_1	G_1		P_1	S_1	T_1
		E_1	T_1			A_1		N_1																A_1				A_1											
						L_1																																	

				2125					2130					2135					2140					2145					2150					2155					2160
S_9	T_9	V_9	V_9	D_9	N_9	T_9	M_9	I_9	L_9	M_9	I_9	V_9	V_9	E_{13}	Y_9	C_9	S_9	E_9	V_9	L_9	S_6	Q_9	E_9	G_9	V_8	S_9	M_9	K_{10}	Y_{10}	K_7	Y_9	M_9	C_9	N_9	G_9	D_9	D_9	L_{10}	I_9
D_4	N_4	T_4	M_4	I_4	L_4	M_4	I_4	V_4	V_4	E_4	Y_4	C_4	S_4	C_1	V_4	M_4	A_4	H_4	E_4	G_4	I_3	E_4	M_4	K_3	Y_3	K_5	Y_5	M_4	C_4	N_4	G_4	D_4	D_4	L_4	I_4	L_5	N_4	A_3	P_4
V_1	V_1	D_1	N_1	T_1	M_1	I_1	L_1	M_1	I_1	V_1	V_1	E_1	Y_1		S_1	E_1	V_1	L_1	S_1	Q_1	L_3	G_1	V_1	S_2	F_1					S_2	C_1	N_1	G_1	D_1	D_1		I_1	V_1	N_1
																					E_1				I_1					M_1									
																					V_1				M_1														

				2165					2170					2175					2180					2185					2190					2195					2200
L_9	N_9	A_9	P_9	D_9	D_9	E_9	I_{10}	P_9	I_9	I_9	Q_9	S_{10}	R_9	F_{13}	K_9	D_7	L_9	F_9	A_9	E_9	C_9	G_9	L_9	N_9	Y_9	D_{10}	F_9	D_9	D_9	V_9	H_9	K_9	S_9	I_9	E_9	T_9	I_9	E_9	Y_9
D_4	E_4	E_4	I_3	P_4	M_4	I_4	Q_4	S_4	R_4	F_4	K_4	N_3	L_4	D_1	A_4	E_4	C_4	G_4	L_4	N_4	Y_4	D_4	F_4	D_5	D_4	V_4	H_4	K_4	E_2	M_4	D_3	T_4	M_4	E_4	Y_4	M_4	S_4	H_4	S_5
A_1	P_1	D_1	V_1	E_1	I_1	P_1		I_1	Q_1	S_1	R_1	F_1	K_1		L_1	S_2	A_1	E_1	C_1	G_1	L_1	N_1	Y_1		F_1		D_1	V_1	H_1	K_1	S_2	I_1	E_1	T_1	M_1	E_1	Y_1	M_1	
			D_1													F_1													K_1										
																													S_1										

				2205					2210					2215					2220					2225					2230					2235					2240
M_9	S_{10}	H_9	S_{12}	F_9	A_9	Q_9	R_9	D_8	G_9	F_9	F_7	I_9	P_9	K_{10}	L_9	K_9	K_9	E_8	S_9	I_6	M_6	A_9	I_7	L_9	E_{10}	W_9	E_{13}	R_7	G_9	D_9	E_9	V_8	M_9	S_{10}	T_9	R_7	S_9	A_{13}	L_9
F_4	A_4	R_3	K_1	D_4	G_4	F_4	F_4	I_4	P_4	K_4	L_4	K_5	K_4	E_4	S_4	M_4	V_4	A_4	M_3	L_4	E_4	W_4	E_5	S_4	G_4	D_4	G_1	I_2	M_4	S_4	T_4	S_5	S_4	A_4	L_4	S_3	A_4	N_1	Y_4
H_1		F_1	A_1	Q_1	R_1	D_1	G_1	G_1	Y_1	I_1	Y_2		L_1		K_1	E_1	S_1	I_1	I_1	M_2	V_4	L_1	M_2	W_1		S_1		V_2	E_1	V_1	M_1	I_1	T_1		S_1	N_3	L_1		A_1
		Q_1						F_1			P_1							D_1	V_1	A_1								S_2								A_1			
																				V_1								D_1											

				2245					2250					2255					2260					2265					2270					2275					2280
N_9	A_9	A_9	Y_9	I_9	E_9	S_9	F_9	G_9	Y_9	D_9	D_9	L_9	M_9	M_8	E_{10}	I_9	E_9	R_7	F_7	A_{13}	V_9	F_9	W_9	A_9	S_9	E_{11}	K_9	G_9	C_9	E_9	Y_9	P_9	L_9	L_8	D_9	S_9	K_9	R_9	V_9
I_4	E_4	S_4	F_4	G_4	Y_4	D_4	D_4	L_4	M_4	T_3	E_4	I_4	E_5	S_4	F_2	A_4	V_4	F_4	W_4	F_1	S_4	E_4	K_3	G_4	C_4	G_2	Y_4	P_4	L_4	L_4	D_4	S_4	K_4	R_4	V_4	E_4	G_4	L_4	Y_4
A_1	Y_1	I_1	E_1	S_1	F_1	G_1	Y_1	D_1	D_1	A_1	M_1	T_1		I_1	Y_2	S_1	Y_1	S_2	Y_2		W_1	A_1	S_2	E_1	K_1	S_1	C_1	E_1	Y_1	P_1	L_1	L_1	D_1	M_1	K_1	R_1	V_1	E_1	G_1
										L_1				V_1				A_1	V_1															S_1					

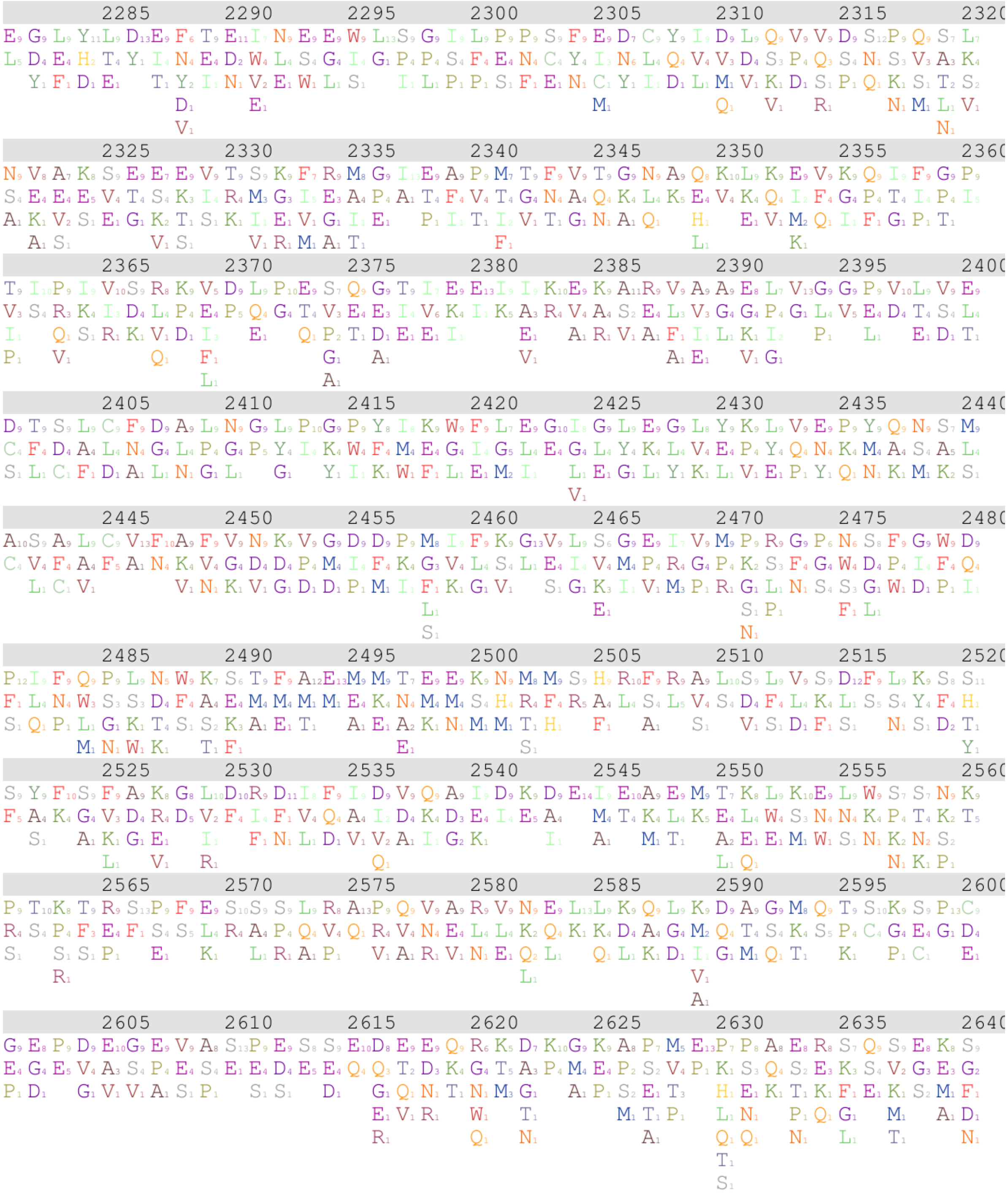

Supplemental Figure 2. UCBSV AA variability determined using datamonkey.org.

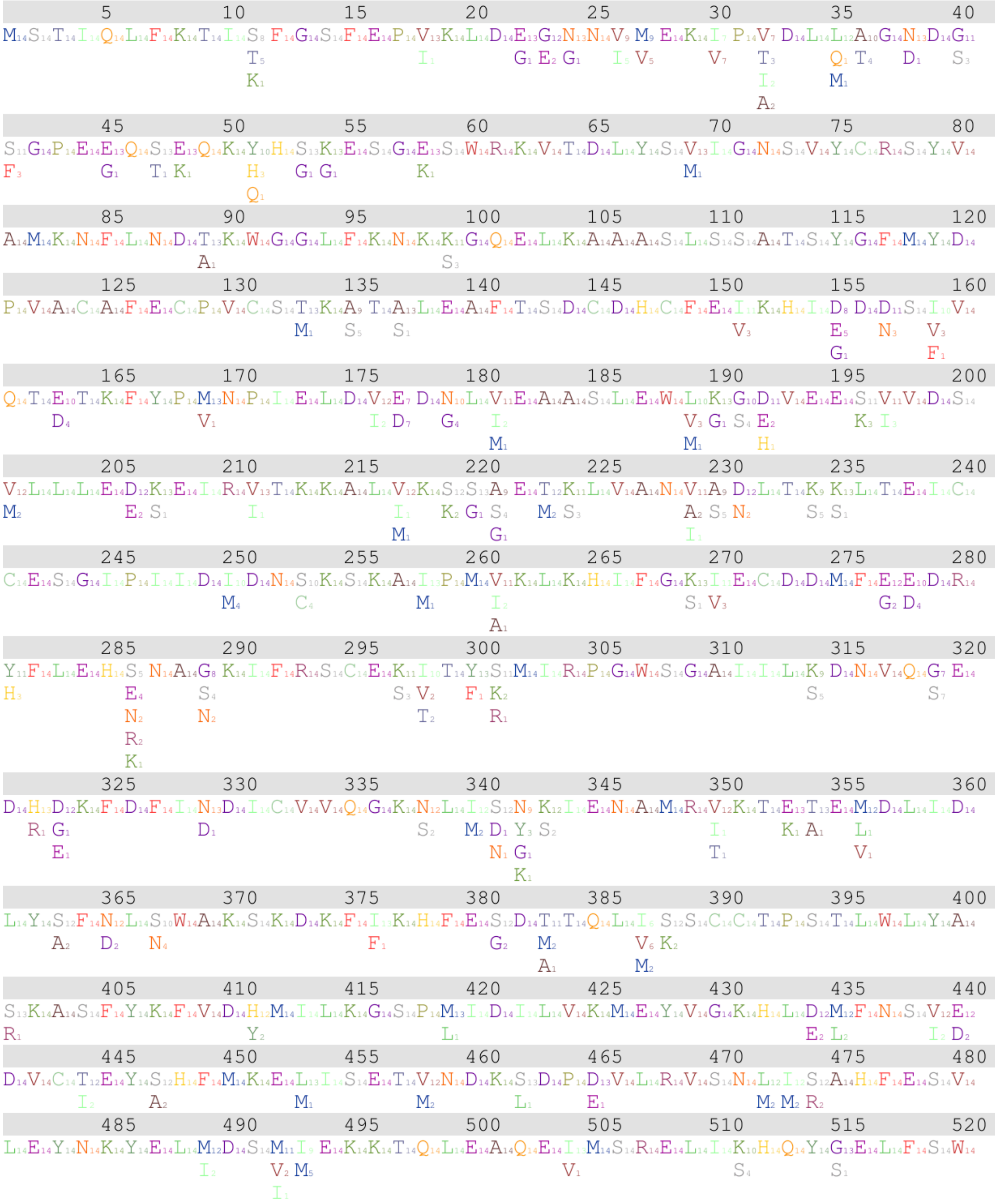

525 530 535 540 545 550 555 560

$R_{14}E_{14}R_{14}L_{11}C_{11}L_{12}K_{12}L_{14}G_{14}M_{14}G_{14}S_{14}S_{14}N_{14}L_{14}Y_{14}T_{14}Y_{14}W_{14}V_{12}E_{14}R_{14}E_{14}E_{14}K_{10}Q_{12}S_{13}E_{14}K_{13}S_{14}S_{10}A_{11}I_{14}S_{14}C_{12}I_{14}I_{14}S_{14}K_{14}P_{14}$

M_{3} Y_{3} M_{2} S_{2} E_{2} S_{2} H_{2} L_{1} S_{1} N_{4} V_{3} F_{2}

Q_{2}

565 570 575 580 585 590 595 600

$G_{14}M_{14}E_{14}M_{14}L_{14}I_{10}N_{12}W_{14}V_{11}S_{14}E_{14}V_{13}C_{14}K_{13}S_{13}K_{14}Y_{14}H_{14}S_{14}L_{14}V_{14}N_{14}C_{14}V_{14}D_{14}S_{13}G_{14}I_{12}L_{13}F_{14}L_{14}W_{14}S_{14}S_{14}M_{11}V_{13}H_{14}L_{14}A_{12}S_{10}$

M_{4} S_{2} A_{2} A_{1} S_{1} N_{1} N_{1} V_{2} M_{1} V_{2} I_{1} M_{2} K_{4}

I_{1} I_{1}

605 610 615 620 625 630 635 640

$V_{12}S_{14}V_{13}Y_{14}G_{14}Y_{14}W_{14}N_{14}L_{14}W_{14}F_{14}S_{14}Q_{14}A_{14}M_{11}C_{14}V_{14}L_{14}F_{14}I_{13}F_{14}L_{14}V_{12}S_{14}N_{14}F_{14}S_{14}S_{7}$ $K_{14}M_{12}V_{14}S_{11}Y_{11}L_{14}K_{14}K_{12}L_{14}I_{14}V_{13}S_{11}$

A_{2} I_{1} V_{2} V_{1} I_{2} G_{5} L_{2} N_{2} F_{3} T_{2} I_{1} N_{3}

L_{1} C_{2} G_{1}

645 650 655 660 665 670 675 680

$E_{14}K_{12}K_{14}L_{14}A_{14}M_{12}K_{14}N_{14}E_{14}E_{12}G_{14}F_{14}V_{13}E_{14}V_{14}Q_{14}G_{14}R_{14}K_{14}E_{14}E_{14}S_{13}F_{14}V_{14}L_{14}K_{14}W_{14}C_{14}A_{14}A_{11}A_{14}T_{14}L_{14}F_{14}L_{14}S_{14}F_{14}L_{14}N_{14}Y_{14}$

S_{2} I_{2} A_{2} I_{1} P_{1} V_{3}

685 690 695 700 705 710 715 720

$D_{14}W_{14}A_{14}V_{14}G_{14}C_{14}V_{13}S_{14}A_{14}I_{14}G_{14}K_{14}M_{14}K_{14}T_{14}M_{12}F_{14}S_{12}A_{14}L_{14}G_{14}P_{14}D_{14}F_{14}M_{12}E_{14}K_{10}Q_{14}D_{14}G_{14}D_{14}D_{14}D_{14}L_{14}K_{13}F_{14}T_{14}T_{14}F_{14}E_{14}$

M_{1} I_{2} G_{2} I_{2} S_{4} S_{1}

725 730 735 740 745 750 755 760

$V_{14}E_{14}I_{14}P_{14}G_{14}D_{14}S_{14}S_{14}S_{14}S_{14}S_{14}A_{14}Q_{14}T_{14}F_{14}G_{14}E_{14}W_{14}I_{12}E_{14}H_{14}C_{14}I_{14}K_{14}F_{13}N_{14}L_{14}V_{14}S_{14}I_{14}E_{14}P_{14}T_{14}T_{14}S_{14}G_{14}P_{14}M_{14}L_{14}T_{14}$

M_{2} Y_{1}

765 770 775 780 785 790 795 800

$L_{14}E_{14}R_{14}G_{14}K_{14}A_{14}N_{14}E_{14}L_{14}A_{14}D_{14}Q_{14}M_{12}N_{14}C_{14}L_{14}N_{14}A_{14}T_{14}D_{14}M_{13}R_{14}V_{14}H_{14}G_{14}G_{14}V_{14}G_{14}T_{14}G_{14}K_{14}S_{14}T_{14}A_{11}L_{14}P_{14}Y_{14}E_{14}L_{14}M_{14}$

I_{2} I_{1} S_{2}

T_{1}

805 810 815 820 825 830 835 840

$S_{14}Y_{14}G_{14}A_{14}V_{14}L_{14}V_{13}C_{14}V_{14}P_{14}T_{14}S_{14}V_{14}L_{14}A_{14}N_{14}A_{14}L_{14}H_{14}E_{14}S_{14}F_{14}M_{14}S_{14}L_{14}F_{14}G_{14}F_{14}D_{14}V_{14}S_{14}L_{14}A_{14}Y_{14}R_{12}G_{13}R_{14}V_{14}S_{14}T_{14}$

M_{1} S_{2} S_{1}

845 850 855 860 865 870 875 880

$G_{14}S_{14}K_{14}P_{14}I_{14}T_{14}I_{14}M_{14}T_{14}Y_{14}G_{14}Y_{14}A_{14}L_{14}N_{14}H_{14}F_{14}H_{14}H_{14}N_{14}P_{14}S_{10}N_{14}L_{14}A_{14}Q_{14}F_{14}Q_{14}F_{14}M_{11}M_{12}M_{14}D_{14}E_{14}V_{14}H_{14}T_{14}F_{14}P_{14}V_{14}$

K_{4} V_{2} L_{2}

I_{1}

885 890 895 900 905 910 915 920

$H_{14}L_{14}N_{14}P_{14}L_{14}F_{14}S_{14}L_{14}L_{14}R_{14}E_{14}L_{14}S_{14}P_{14}D_{14}K_{14}K_{14}M_{12}I_{14}K_{14}T_{14}S_{14}A_{14}T_{14}H_{14}V_{14}G_{14}H_{14}N_{13}V_{14}D_{14}L_{14}S_{14}T_{14}N_{14}H_{14}K_{14}V_{14}D_{14}M_{12}$

I_{2} S_{1} I_{2}

925 930 935 940 945 950 955 960

$H_{14}T_{14}L_{14}E_{14}M_{9}$ $M_{14}D_{14}V_{14}K_{14}K_{14}W_{14}A_{14}E_{14}L_{14}Q_{14}G_{14}T_{14}S_{14}V_{14}F_{14}G_{14}D_{14}V_{14}T_{14}K_{12}E_{14}P_{12}G_{14}N_{14}V_{13}L_{14}V_{13}F_{14}V_{14}A_{14}S_{14}Y_{14}S_{14}D_{13}V_{14}$

V_{3} S_{2} H_{1} I_{1} I_{1} E_{1}

L_{2} S_{1}

965 970 975 980 985 990 995 1000

$D_{14}V_{14}C_{14}A_{13}E_{12}K_{14}L_{14}K_{14}D_{14}K_{14}G_{14}F_{14}P_{14}V_{14}I_{12}K_{14}V_{14}D_{14}G_{14}S_{12}N_{14}F_{14}S_{14}K_{14}N_{14}T_{14}E_{14}V_{14}Q_{14}K_{14}M_{14}V_{14}D_{14}G_{8}$ $M_{13}Q_{10}G_{14}E_{14}V_{14}K_{14}$

S_{1} D_{2} M_{2} R_{2} E_{4} L_{1} R_{2}

A_{2} K_{1}

M_{1}

1005 1010 1015 1020 1025 1030 1035 1040

$F_{14}I_{12}V_{14}A_{14}T_{14}N_{14}I_{14}I_{14}E_{14}N_{14}G_{14}V_{14}T_{14}L_{14}D_{14}V_{14}D_{14}V_{14}V_{14}V_{13}D_{14}F_{14}G_{14}E_{14}R_{14}M_{14}S_{14}P_{14}N_{14}L_{14}C_{14}S_{14}E_{14}D_{13}S_{14}C_{14}I_{14}L_{14}M_{14}Q_{14}$

M_{2} I_{1} E_{1}

1045 1050 1055 1060 1065 1070 1075 1080

$S_{14}Q_{14}S_{14}I_{14}S_{14}Q_{14}A_{14}E_{14}S_{14}K_{14}Q_{14}S_{14}F_{14}G_{14}S_{14}V_{14}G_{14}S_{14}M_{14}K_{14}S_{13}G_{14}S_{14}V_{14}Y_{14}K_{14}F_{14}G_{14}S_{14}E_{12}T_{14}L_{14}P_{14}D_{14}S_{14}M_{14}S_{14}S_{9}$ $S_{14}V_{14}$

K_{1} D_{2} N_{5}

1085 1090 1095 1100 1105 1110 1115 1120

$G_{14}S_{14}T_{14}E_{14}S_{14}A_{14}L_{14}I_{14}C_{14}F_{14}A_{14}Y_{14}G_{14}L_{14}K_{14}P_{14}V_{14}V_{14}D_{14}D_{14}V_{14}D_{14}M_{14}G_{14}S_{14}V_{14}S_{14}S_{13}V_{14}T_{14}Q_{14}S_{14}Q_{14}A_{14}L_{14}T_{14}A_{14}S_{14}M_{14}F_{14}$

G_{1}

```
1125 1130 1135 1140 1145 1150 1155 1160
E14 A14 N14 Y14 M14 F14 T14 A13 H14 L14 V14 D14 K14 Q14 G14 F14 M14 P14 S14 P14 V14 F14 E14 L14 M14 K14 N7 L14 L14 L14 H14 T14 D14 A14 V14 G14 V13 S14 S14 T14
  [1128: T1] [1147: S7] [1157: I1]

1165 1170 1175 1180 1185 1190 1195 1200
Y14 L14 A14 T14 N14 M14 S14 G14 W14 S13 S14 L14 K14 E14 Y14 I13 S12 I1 D14 D14 S10 S14 R14 H14 V14 Q14 E14 V13 Q14 I14 P14 W14 Y14 C14 S14 D14 M14 S14 D14 D14
  [1170: K1] [1176: V1] [1177: K2] [1181: N4] [1188: I1]

1205 1210 1215 1220 1225 1230 1235 1240
F14 I14 V12 K14 L14 A14 E14 C14 V14 K14 A14 A14 K14 P14 K14 S14 Q14 C14 G14 Y14 K14 V14 D14 N14 V14 D14 F12 H14 T14 V14 A14 H14 K14 M14 S14 V13 G14 E14 S14 N14
  [1203: I2] [1227: L2] [1236: M1]

1245 1250 1255 1260 1265 1270 1275 1280
M14 D14 E14 S14 S13 A14 L14 V14 A13 T14 I14 L14 D14 E14 V14 K14 Q14 W14 S14 D14 G14 I14 T14 Y14 H14 S14 S14 T14 P14 S14 N14 K14 S14 L14 M14 S14 L14 M14 V14 G14
  [1245: R1] [1249: S1]

1285 1290 1295 1300 1305 1310 1315 1320
W14 I14 P14 S14 K14 A14 E14 K14 T14 K14 E14 I14 L14 D14 N14 R12 I12 Q14 R14 L14 E14 L14 L14 L14 N14 Q14 L14 N14 G14 V14 S14 G14 I13 D14 D14 Y14 E14 S14 L14 V14
  [1296: S2] [1297: V2] [1313: V1]

1325 1330 1335 1340 1345 1350 1355 1360
R14 F14 F14 S14 E14 N14 P14 H14 S14 A14 E14 Y14 L14 E14 S14 Q14 C14 A14 S14 D14 Y14 I14 E14 E14 K14 V14 M14 S12 V14 K14 S14 N12 Y14 D14 K14 S12 L13 I13 L14 G14
  [1348: N2] [1352: S2] [1356: P2] [1357: M1] [1358: M1]

1365 1370 1375 1380 1385 1390 1395 1400
M13 V14 G14 L14 A14 V14 A14 T14 G14 T14 F14 A14 Y14 W14 Y14 M14 S14 S14 S13 A14 A14 V13 E14 L14 V14 E14 K14 Q14 A14 K14 H14 K14 Y14 N14 S14 D14 K14 R12 T14 G14
  [1361: V1] [1379: D1] [1382: M1] [1398: S2]

1405 1410 1415 1420 1425 1430 1435 1440
S14 L13 M12 F14 D12 M14 D14 D14 Q13 E14 T14 Y14 E14 N12 F14 G14 P10 E14 Y14 T14 D14 D14 V14 I11 S14 A13 K14 M14 T13 K14 A14 Q14 K14 E14 S14 D12 S14 K13 K14 K14
  [1403: L2] [1405: E2] [1409: S1] [1414: S2] [1417: A2, S2] [1424: M3] [1426: G1] [1429: A1] [1436: E2] [1438: S1]

1445 1450 1455 1460 1465 1470 1475 1480
G14 W14 K14 A14 G14 K14 M10 N14 R14 P14 M14 S14 V13 F14 H14 Q14 L14 Y14 G14 V14 N14 P14 L14 E14 F14 D14 E14 V14 V12 M14 S12 V14 G14 K14 L14 E14 T13 E14 P14 W14
  [1447: V4] [1453: I1] [1469: I2] [1471: R2] [1477: A1]

1485 1490 1495 1500 1505 1510 1515 1520
D14 V14 K14 E14 L14 N14 V13 D13 A12 M14 M12 I12 E14 L14 D14 D14 D14 Y14 H14 I14 L14 R14 D14 D14 R12 M14 F13 G14 K14 K12 V14 S14 L14 A14 F14 K11 K14 E14 G14 A12
  [1487: I1] [1488: G1] [1489: G2] [1491: L2] [1505: S2] [1507: Y1] [1510: T2] [1516: S3] [1520: S2]

1525 1530 1535 1540 1545 1550 1555 1560
D10 E14 E14 T14 I14 V14 N13 L14 T14 P14 H14 R14 S14 K14 M14 A8 S14 S14 M14 S14 L14 A14 P14 M14 G14 F14 P14 E14 E14 E14 G14 S14 W14 S14 Q14 T14 G14 A14 P14 L10
  [1521: H2, G1, N1] [1527: S1] [1536: T6] [1560: V3, M1]

1565 1570 1575 1580 1585 1590 1595 1600
M11 K10 K14 I14 K8 K10 E14 D12 E14 V12 E14 V14 Q14 V14 A14 K14 P14 E14 P12 T14 N14 P14 Y14 D14 H14 I12 L14 V14 S14 L14 G14 S14 A14 H13 L14 G14 T14 R14 V14 L14
  [1561: V2, T1] [1562: S3, Q1] [1564: T1] [1565: E4, S2] [1566: N2, T2] [1568: E2] [1570: I2] [1579: L1, S1] [1586: V2] [1594: R1]

1605 1610 1615 1620 1625 1630 1635 1640
N14 C14 F14 F14 H14 G14 S12 K14 C14 V13 I13 P14 Y14 H14 L14 A14 E14 K14 G14 D14 S14 E14 E14 S12 L14 V10 I13 A14 T14 T14 S14 G14 Q14 F14 D14 F14 G14 P14 M13 K13
  [1607: A1, T1] [1610: I1] [1624: P2] [1626: I4] [1639: L1] [1640: E1]

1645 1650 1655 1660 1665 1670 1675 1680
N14 I14 K14 C14 S12 K14 I7 T14 D14 Y14 D14 I10 T14 I14 C14 P13 L14 P14 N14 D14 V14 Q14 P14 F14 R14 S13 K14 I14 V14 M14 R14 E14 P14 K14 L14 G14 E14 E14 V14 V14
  [1645: K2] [1647: V7] [1652: V3, M1] [1656: S1] [1666: A1]

1685 1690 1695 1700 1705 1710 1715 1720
I14 V14 C12 F14 T12 S14 I13 N14 G14 K14 I14 V14 M14 K14 V13 S14 D14 K14 S14 T12 T14 Y14 P14 A14 G14 G14 Q14 F14 A14 H14 L14 W14 A14 Y14 K11 Y14 D14 G14 Q14 P14
  [1683: Y2] [1685: A2] [1695: A1] [1700: A2] [1715: N3]

1725 1730 1735 1740 1745 1750 1755 1760
G14 D14 C14 G14 G14 P14 I14 V14 A14 T14 V14 D14 Q14 K14 V14 V14 G14 F14 H14 S14 G14 V14 I14 S14 N14 S14 S14 E11 E14 K14 L14 R14 A14 V14 Y14 T14 P14 V14 N14 Q14
  [1748: G3]
```

1765 1770 1775 1780 1785 1790 1795 1800

$E_{14}L_{14}L_{12}N_{14}C_{12}I_{10}S_{14}G_{12}D_{14}I_{11}Q_{14}M_{14}T_{14}D_{14}F_{14}W_{14}T_{14}F_{14}N_{14}P_{14}D_{14}L_{14}V_{14}E_{14}W_{14}N_{14}S_{14}V_{14}A_{14}S_{14}V_{14}S_{14}T_{14}F_{14}F_{14}P_{14}M_{14}T_{14}K_{14}A_{14}$

M_{2} Y_{2} V_{4} S_{2}

1805 1810 1815 1820 1825 1830 1835 1840

$I_{14}N_{14}T_{14}I_{12}T_{14}V_{14}Q_{14}A_{14}N_{11}E_{14}G_{14}E_{14}E_{14}L_{13}I_{14}D_{14}G_{14}N_{14}L_{14}M_{14}I_{12}V_{14}G_{14}Y_{14}V_{14}N_{14}R_{14}E_{14}V_{14}Y_{14}H_{14}N_{14}H_{14}V_{13}I_{13}K_{14}G_{14}K_{12}S_{14}E_{14}$

M_{2} D_{3} S_{1} M_{2} I_{1} S_{2}

1845 1850 1855 1860 1865 1870 1875 1880

$S_{14}F_{14}M_{10}S_{13}Y_{14}C_{14}E_{14}Q_{14}F_{14}P_{14}N_{12}C_{14}A_{14}F_{14}T_{14}K_{14}E_{12}L_{14}R_{12}D_{14}Q_{13}Y_{14}L_{14}P_{14}S_{14}I_{11}L_{14}S_{14}K_{14}P_{14}A_{14}F_{14}S_{14}K_{14}G_{14}L_{14}L_{14}K_{14}Y_{14}N_{14}$

T_{4} K_{1} S_{2} D_{2} S_{2} K_{1} V_{3}

1885 1890 1895 1900 1905 1910 1915 1920

$E_{14}P_{14}V_{14}R_{13}V_{14}G_{14}S_{10}V_{11}N_{10}F_{14}P_{9}C_{14}L_{14}M_{12}R_{14}A_{14}Y_{14}L_{14}K_{12}V_{14}E_{14}E_{8}M_{14}F_{14}E_{13}N_{6}L_{14}G_{14}F_{14}L_{12}K_{7}E_{14}A_{14}G_{14}P_{14}Q_{14}W_{14}D_{14}P_{14}I_{14}$

Q_{1} L_{4} M_{2} D_{4} S_{5} I_{2} N_{2} G_{4} K_{1} D_{4} R_{2} E_{5}

L_{1} T_{2} T_{2} G_{2}

A_{2}

1925 1930 1935 1940 1945 1950 1955 1960

$E_{14}I_{14}L_{14}E_{9}D_{14}L_{14}N_{14}K_{14}K_{14}A_{14}A_{14}M_{14}G_{14}A_{14}L_{14}Y_{14}Q_{14}G_{14}K_{14}K_{14}Q_{14}D_{14}W_{14}L_{13}K_{14}S_{14}M_{12}E_{14}P_{14}A_{13}D_{14}F_{14}I_{14}T_{10}A_{14}V_{14}R_{14}E_{14}S_{14}F_{14}$

D_{5} F_{1} I_{2} V_{1} M_{4}

1965 1970 1975 1980 1985 1990 1995 2000

$K_{14}H_{14}L_{14}A_{14}G_{14}G_{14}D_{14}V_{14}G_{14}I_{14}W_{14}S_{14}G_{14}S_{14}L_{14}K_{14}A_{14}E_{14}L_{14}S_{14}P_{12}V_{12}E_{10}K_{14}V_{14}L_{14}E_{14}Q_{14}K_{14}T_{14}S_{12}V_{14}F_{14}T_{14}G_{14}A_{14}P_{14}I_{13}D_{14}L_{14}$

T_{1} I_{2} K_{4} R_{2}

S_{1}

2005 2010 2015 2020 2025 2030 2035 2040

$L_{14}L_{14}G_{14}G_{14}K_{14}I_{12}L_{14}V_{14}D_{14}N_{14}F_{14}N_{14}H_{14}F_{14}F_{14}Y_{14}F_{14}N_{14}H_{14}L_{14}K_{14}G_{14}P_{14}W_{14}T_{14}V_{14}G_{14}I_{14}N_{14}K_{14}F_{14}N_{14}K_{14}G_{14}W_{14}D_{14}S_{14}L_{14}A_{14}S_{12}$

M_{2} K_{2}

2045 2050 2055 2060 2065 2070 2075 2080

$Y_{13}F_{14}N_{12}H_{14}S_{12}W_{14}N_{14}F_{14}M_{13}D_{14}C_{14}D_{14}G_{14}S_{14}S_{14}F_{14}D_{14}T_{14}S_{14}L_{14}A_{14}P_{14}I_{14}L_{14}F_{14}Q_{14}L_{14}V_{14}C_{14}H_{14}M_{14}R_{14}E_{14}K_{14}F_{14}G_{14}N_{14}F_{14}D_{14}D_{14}$

N_{1} D_{2} N_{2} T_{1}

2085 2090 2095 2100 2105 2110 2115 2120

$I_{13}E_{14}S_{12}A_{12}A_{14}L_{14}R_{14}N_{14}L_{14}Y_{14}T_{14}Q_{14}M_{14}V_{14}Y_{14}T_{14}P_{14}I_{14}L_{14}T_{14}I_{12}D_{14}G_{14}Y_{14}I_{14}T_{8}K_{14}K_{14}H_{14}R_{14}G_{14}N_{14}N_{14}S_{14}G_{14}Q_{14}P_{14}S_{14}T_{14}V_{14}$

V_{1} K_{2} T_{2} A_{4}

M_{1}

V_{1}

2125 2130 2135 2140 2145 2150 2155 2160

$V_{14}D_{14}N_{14}T_{14}I_{12}M_{11}L_{14}M_{14}I_{14}V_{14}V_{14}E_{14}Y_{14}C_{14}K_{14}A_{14}V_{14}M_{14}E_{14}S_{12}E_{14}G_{14}S_{13}T_{4}M_{14}Q_{14}F_{14}K_{14}Y_{14}M_{14}C_{14}N_{14}G_{14}D_{14}D_{14}L_{14}I_{12}L_{14}N_{14}V_{14}$

M_{2} I_{3} T_{2} K_{1} V_{4} M_{2}

I_{2}

E_{2}

A_{2}

2165 2170 2175 2180 2185 2190 2195 2200

$P_{14}D_{12}D_{14}E_{14}V_{14}S_{12}M_{14}I_{13}Q_{14}S_{14}S_{14}F_{14}S_{14}E_{11}L_{14}F_{14}S_{14}E_{14}C_{14}G_{14}L_{14}D_{14}Y_{14}N_{13}F_{14}D_{14}D_{14}V_{14}H_{14}K_{14}S_{14}M_{14}E_{14}T_{14}I_{14}E_{14}Y_{14}M_{14}S_{14}H_{14}$

N_{2} N_{2} V_{1} D_{2} T_{1}

N_{1}

2205 2210 2215 2220 2225 2230 2235 2240

$S_{14}F_{14}M_{14}L_{13}K_{14}D_{14}G_{10}V_{12}Y_{14}I_{14}P_{14}K_{14}L_{14}K_{14}K_{12}E_{14}S_{14}I_{11}V_{14}A_{14}I_{14}L_{14}E_{14}W_{14}E_{14}S_{14}G_{14}D_{14}E_{14}I_{10}M_{14}R_{12}T_{14}R_{12}S_{14}A_{14}L_{14}N_{14}A_{14}A_{14}$

M_{1} D_{4} L_{1} S_{2} V_{1} M_{4} S_{2} S_{2}

M_{1} M_{1}

2245 2250 2255 2260 2265 2270 2275 2280

$Y_{14}I_{13}E_{14}S_{14}Y_{14}G_{14}Y_{14}E_{8}D_{14}L_{13}M_{14}V_{11}E_{14}I_{14}E_{14}R_{14}Y_{14}A_{14}V_{14}F_{14}W_{14}A_{14}T_{8}E_{13}K_{14}G_{14}C_{14}E_{14}Y_{14}P_{14}L_{14}L_{14}D_{14}S_{14}K_{14}R_{14}V_{14}E_{14}G_{14}L_{14}$

D_{6} P_{1} I_{2} A_{6} G_{1}

T_{1}

M_{1}

2285 2290 2295 2300 2305 2310 2315 2320

$Y_{14}K_{10}D_{14}D_{14}H_{11}T_{14}D_{14}I_{14}N_{14}E_{14}E_{14}W_{14}L_{14}M_{6}G_{14}I_{14}L_{14}P_{14}P_{14}S_{14}F_{14}E_{14}H_{14}C_{14}Y_{14}V_{14}D_{14}T_{10}Q_{14}T_{13}K_{12}D_{14}L_{12}S_{13}G_{8}S_{9}E_{12}K_{10}P_{12}E_{14}$

S_{4} Y_{2} V_{3} M_{4} I_{1} E_{2} V_{2} K_{1} E_{4} G_{2} D_{2} Q_{2} L_{2}

N_{1} I_{2} S_{2} E_{2} E_{2}

S_{2} K_{1}

T_{1}

2325 2330 2335 2340 2345 2350 2355 2360

$L_{13}S_{13}I_{5}E_{12}S_{13}H_{10}D_{13}G_{11}V_{6}P_{5}Q_{11}M_{13}Q_{14}M_{14}K_{14}F_{14}P_{14}V_{14}T_{14}F_{14}V_{14}T_{14}G_{14}N_{14}L_{12}G_{14}K_{14}L_{14}A_{14}E_{14}V_{14}K_{13}S_{14}I_{14}L_{14}G_{14}I_{14}A_{13}N_{11}D_{14}$

F_{1} G_{1} V_{3} G_{2} N_{1} C_{2} N_{1} S_{3} T_{6} S_{5} R_{3} L_{1} F_{2} S_{1} S_{1} S_{3}

T_{3} Y_{2} A_{1} L_{2}

P_{2} M_{1} T_{2}

A_{1}

2365 2370 2375 2380 2385 2390 2395 2400

$V_{14}M_{9}A_{14}K_{8}N_{14}I_{14}D_{14}L_{14}P_{14}E_{14}V_{14}Q_{14}G_{14}T_{14}P_{14}D_{11}E_{14}I_{13}V_{13}S_{11}K_{14}K_{14}A_{14}Q_{14}L_{13}A_{14}V_{14}K_{14}M_{14}T_{14}N_{14}S_{12}P_{14}V_{14}L_{14}V_{14}E_{14}D_{14}T_{13}C_{14}$

V_{5} S_{5} E_{2} V_{1} A_{1} K_{2} F_{1} G_{2} A_{1}

R_{1} V_{1} M_{1}

2405 2410 2415 2420 2425 2430 2435 2440

$L_{14}C_{14}F_{14}N_{14}A_{14}F_{14}N_{14}G_{14}L_{14}P_{14}G_{14}P_{14}Y_{14}I_{13}K_{14}W_{14}F_{14}L_{14}K_{14}E_{14}L_{14}G_{14}L_{14}E_{8}G_{14}V_{14}V_{14}K_{12}M_{14}L_{14}S_{14}A_{14}F_{14}G_{8}D_{14}K_{14}S_{14}A_{14}Y_{14}A_{14}$

M_{1} D_{6} S_{2} E_{4}

S_{2}

2445 2450 2455 2460 2465 2470 2475 2480

$L_{14}C_{14}T_{14}F_{14}A_{14}Y_{14}V_{13}H_{14}N_{13}E_{14}L_{7}S_{14}D_{13}P_{14}I_{11}V_{14}F_{14}K_{14}G_{14}V_{14}V_{14}N_{13}G_{14}E_{14}I_{14}V_{14}P_{14}P_{14}R_{14}G_{14}N_{12}N_{14}G_{14}F_{14}G_{14}W_{14}D_{14}P_{14}M_{9}F_{14}$

A_{1} S_{1} S_{7} E_{1} V_{3} K_{1} K_{2} I_{5}

2485 2490 2495 2500 2505 2510 2515 2520

$K_{14}P_{14}D_{14}G_{9}C_{14}G_{7}C_{14}T_{14}F_{14}A_{14}E_{14}M_{14}P_{9}S_{14}S_{11}I_{13}K_{14}N_{14}D_{13}F_{14}S_{14}H_{14}S_{14}S_{14}S_{13}A_{14}L_{14}E_{14}K_{14}V_{14}K_{14}L_{11}F_{10}L_{14}D_{14}N_{14}L_{14}V_{10}V_{14}K_{12}$

E_{5} S_{6} S_{5} N_{2} M_{3} E_{1} K_{1} S_{3} Y_{4} M_{4} S_{2}

E_{1} G_{1} N_{1}

2525 2530 2535 2540 2545 2550 2555 2560

$Q_{14}E_{14}E_{12}K_{12}K_{11}A_{13}K_{7}V_{13}A_{14}L_{14}T_{14}I_{14}D_{12}V_{14}Q_{14}A_{13}L_{14}N_{12}Q_{14}E_{14}E_{14}M_{9}E_{14}A_{12}E_{14}I_{14}T_{12}A_{14}L_{14}K_{14}K_{14}L_{13}W_{14}K_{9}D_{14}N_{14}G_{14}P_{14}T_{14}S_{13}$

K_{2} Q_{1} S_{3} T_{1} S_{6} M_{1} E_{2} V_{1} D_{2} V_{4} E_{1} A_{1} M_{1} S_{5} G_{1}

N_{1} G_{1} I_{1} T_{1} S_{1}

2565 2570 2575 2580 2585 2590 2595 2600

$T_{14}R_{14}S_{14}P_{12}F_{14}E_{14}A_{14}S_{13}S_{14}L_{14}S_{14}A_{14}P_{12}Q_{14}V_{14}E_{14}R_{14}V_{14}N_{14}E_{14}L_{14}L_{14}Q_{14}K_{12}L_{14}K_{14}D_{13}E_{14}G_{14}L_{14}Q_{14}T_{14}K_{14}K_{14}S_{14}P_{14}C_{14}G_{14}E_{14}P_{14}$

S_{2} K_{1} S_{2} Q_{2} N_{1}

2605 2610 2615 2620 2625 2630 2635 2640

$D_{14}D_{14}G_{14}E_{14}V_{13}V_{13}D_{13}D_{14}D_{14}S_{14}D_{12}D_{13}G_{12}N_{11}N_{12}Q_{14}S_{14}S_{13}G_{12}K_{14}E_{12}V_{12}V_{8}D_{12}E_{14}S_{11}Q_{14}N_{13}N_{10}Q_{12}Q_{13}P_{12}S_{12}K_{13}P_{10}K_{13}F_{13}K_{14}I_{13}S_{14}$

M_{1} M_{1} V_{1} N_{2} N_{1} D_{1} D_{3} K_{1} F_{1} E_{2} K_{2} I_{4} H_{1} N_{2} D_{1} D_{4} K_{2} H_{1} L_{1} K_{2} S_{1} S_{2} S_{1} L_{1} V_{2}

E_{1} D_{1} N_{1} G_{1} S_{1} L_{1} L_{1}

Q_{1}

2645 2650 2655 2660 2665 2670 2675 2680

$G_{14}D_{14}G_{13}S_{11}A_{10}I_{9}S_{12}R_{14}D_{14}D_{14}I_{14}D_{14}K_{13}I_{14}P_{14}T_{14}N_{13}A_{14}L_{14}E_{14}I_{14}K_{14}K_{14}T_{14}F_{14}K_{14}P_{14}P_{14}K_{10}V_{14}S_{14}Q_{14}S_{14}A_{14}Y_{14}I_{14}W_{14}I_{14}P_{14}R_{14}$

E_{1} N_{3} T_{4} V_{5} K_{2} S_{1} S_{1} S_{4}

2685 2690 2695 2700 2705 2710 2715 2720

$S_{14}Q_{14}S_{14}D_{14}N_{14}L_{14}T_{14}P_{14}D_{14}V_{14}I_{13}Q_{14}N_{13}F_{14}L_{14}A_{14}Y_{14}M_{10}P_{14}P_{14}S_{14}H_{13}A_{14}M_{14}D_{14}N_{14}Q_{14}L_{14}A_{14}S_{14}G_{14}I_{13}E_{14}V_{14}E_{14}N_{14}W_{14}A_{14}I_{12}E_{14}$

M_{1} S_{1} V_{4} Y_{1} V_{1} V_{2}

M_{1}

2725 2730 2735 2740 2745 2750 2755 2760

$V_{14}A_{14}K_{14}A_{14}Y_{14}G_{14}V_{14}N_{14}M_{14}Q_{14}E_{14}F_{14}Y_{14}R_{13}T_{14}V_{10}L_{14}P_{14}A_{14}W_{14}M_{10}V_{14}N_{14}C_{14}M_{14}V_{14}N_{14}G_{14}T_{14}S_{14}D_{14}E_{14}S_{14}K_{14}N_{14}E_{14}K_{14}S_{14}W_{14}R_{14}$

H_{1} I_{4} I_{4}

2765 2770 2775 2780 2785 2790 2795 2800

$A_{14}V_{14}E_{14}L_{14}N_{14}S_{14}Q_{14}G_{14}E_{14}D_{14}V_{14}D_{14}D_{14}F_{14}E_{14}Y_{14}P_{14}M_{14}E_{14}P_{14}M_{14}Y_{14}K_{13}F_{14}A_{14}L_{14}P_{14}T_{14}M_{14}S_{14}K_{14}V_{11}M_{14}S_{14}N_{14}F_{14}S_{14}S_{14}Q_{14}A_{14}$

S_{1} I_{3}
